# *In situ* synthesis of degradable polymer prodrug nanoparticles†

**DOI:** 10.1039/d4sc07746f

**Published:** 2025-01-07

**Authors:** Chen Zhu, Hannah Beauseroy, Julie Mougin, Maëlle Lages, Julien Nicolas

**Affiliations:** a Université Paris-Saclay, CNRS, Institut Galien Paris-Saclay 91400 Orsay France julien.nicolas@universite-paris-saclay.fr +33-180006081

## Abstract

The *in situ* synthesis of degradable polymer prodrug nanoparticles is still a challenge to be met, which would make it possible to remedy both the shortcomings of traditional formulation of preformed polymers (*e.g.*, low nanoparticle concentrations) and those of the physical encapsulation of drugs (*e.g.*, burst release and poor drug loadings). Herein, through the combination of radical ring-opening polymerization (rROP) and polymerization-induced self-assembly (PISA) under appropriate experimental conditions, we report the successful preparation of high-solid content, degradable polymer prodrug nanoparticles, exhibiting multiple drug moieties covalently linked to a degradable vinyl copolymer backbone. Such a rROPISA process relied on the chain extension of a biocompatible poly(ethylene glycol)-based solvophilic block with a mixture of lauryl methacrylate (LMA), cyclic ketene acetal (CKA) and drug-bearing methacrylic esters by reversible addition fragmentation chain transfer (RAFT) copolymerization at 20 wt% solid content. This novel approach was exemplified with two different CKA monomers and two different anticancer drugs, namely paclitaxel and gemcitabine, to demonstrate its versatility. After transferring to water, remarkably stable aqueous suspensions of core-degradable polymer prodrug nanoparticles, 56–225 nm in diameter, with tunable amounts of CKA units (7–26 mol%) and drug loadings of up to 33 wt% were obtained. The incorporation of ester groups in the copolymers was demonstrated by hydrolytic degradation of both the copolymers and the nanoparticles under accelerated conditions. The nanoparticles showed significant cytotoxicity against A549 cells, used as a lung cancer model. Fluorescence labeling of the solvophilic block also enabled effective monitoring of cell internalization by confocal microscopy, with potential for theranostic applications.

## Abstract 2

The synthesis of aqueous suspensions of degradable polymer prodrug nanoparticles with high drug loadings, hydrolitic degradation and cytotoxicity against cancer cells was achieved by radical ring-opening polymerization-induced self-assembly.

## Graphical abstract

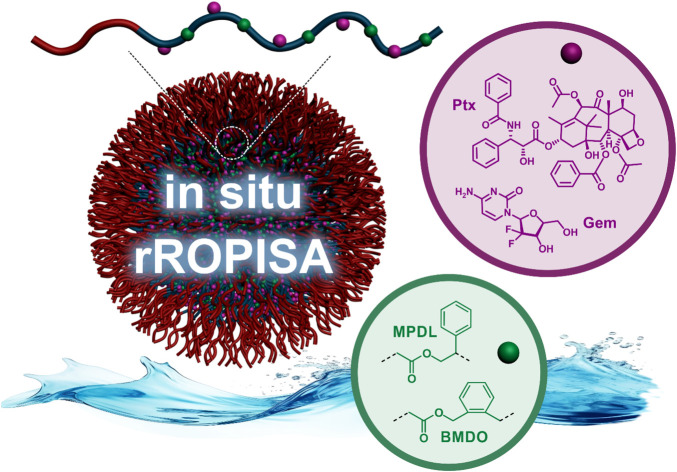

## Introduction

Drug-loaded polymer nanoparticles are being extensively studied for use in a wide range of diseases, such as cancer.^1–3^ They are exclusively obtained by formulating pre-synthesized degradable polymers in the presence of drugs using emulsification methods such as nanoprecipitation or emulsification-solvent evaporation.^4^ Yet, this preparation process has significant shortcomings, such as the production of low-concentration nanoparticles with low drug loadings (typically 1–5 wt%) and the early and abrupt release of the drug post-administration (*i.e.*, burst release), which induces prohibitive toxicity and reduces therapeutic efficacy. Compatibility problems between the drug and the polymer matrix may also arise, which can affect the colloidal stability of the nanoparticles.

Some of these issues can be alleviated using polymer prodrugs (also called polymer drug conjugates), in which the drugs are chemically bound to the polymer.^5–7^ The resulting polymer prodrug nanocarriers generally exhibit sustained drug release and higher drug loadings, leading to greater therapeutic efficacy compared to the traditional drug-loaded counterparts. Nevertheless, these systems still require formulation methods based on preformed polymers for their preparation. There is therefore an urgent need to develop a robust two-in-one strategy to generate *in situ* degradable polymer prodrug nanoparticles for drug delivery applications.

Over the past few decades, polymerization-induced self-assembly (PISA) has become a well-known one-step synthetic procedure for the manufacture of surfactant-free high-solids block copolymer nanoparticles.^8–11^ The robustness and versatility of PISA have made it possible to design nano-objects for many applications,^11–15^ such as biomedical applications with potential use in drug delivery.^12,14,16,17^ For instance, PISA processes have been developed for the *in situ* encapsulation of enzymes^18,19^ and proteins into polymer nanoparticles,^20^ as well as the synthesis of polymer–protein bioconjugate nanoparticles.^21–24^ Physical encapsulation of drugs^25,26^ and their grafting onto polymer nanoparticles post-PISA^27–29^ have also been studied. However, all these systems are based on non-degradable vinyl polymers, which could lead to cumulative toxicity when administered *in vivo*, thus compromising their potential for clinical translation. Although some studies has reported aqueous suspensions of degradable nano-objects by PISA, either by copolymerization of vinyl monomers with cyclic ketene acetals (CKA), cyclic allylic sulfides or thionolactones, *via* radical ring-opening polymerization-induced self-assembly (rROPISA),^30–33^ or by ring-opening polymerization of *N*-carboxyanhydrides,^34^ all these examples were more fundamental and did not focus on drug loading and delivery.

Unlike the formulation of pre-synthesized rROP-derived (co)polymers into nanoparticles,^35–40^ the rROPISA process based on CKAs produces core-degradable vinyl polymer nanoparticles *in situ*, either in organic^41,42^ or aqueous media,^43^ through the chain extension of a solvophilic block by copolymerizing vinyl monomers and CKAs. To generate aqueous suspensions of degradable nanoparticles, while avoiding early hydrolysis of CKA monomers in water, the rROPISA process is performed in DMF followed by transfer of the obtained nanoparticles to water *via* dialysis.^43^ This strategy preserves the integrity of CKAs, while maintaining the colloidal properties of the nanoparticles during their transfer to water. This process also makes it possible to obtain nanoparticles that can be degraded by hydrolysis and exhibit high cytocompatibility towards various healthy cell lines.^43^

**Fig. 1:**
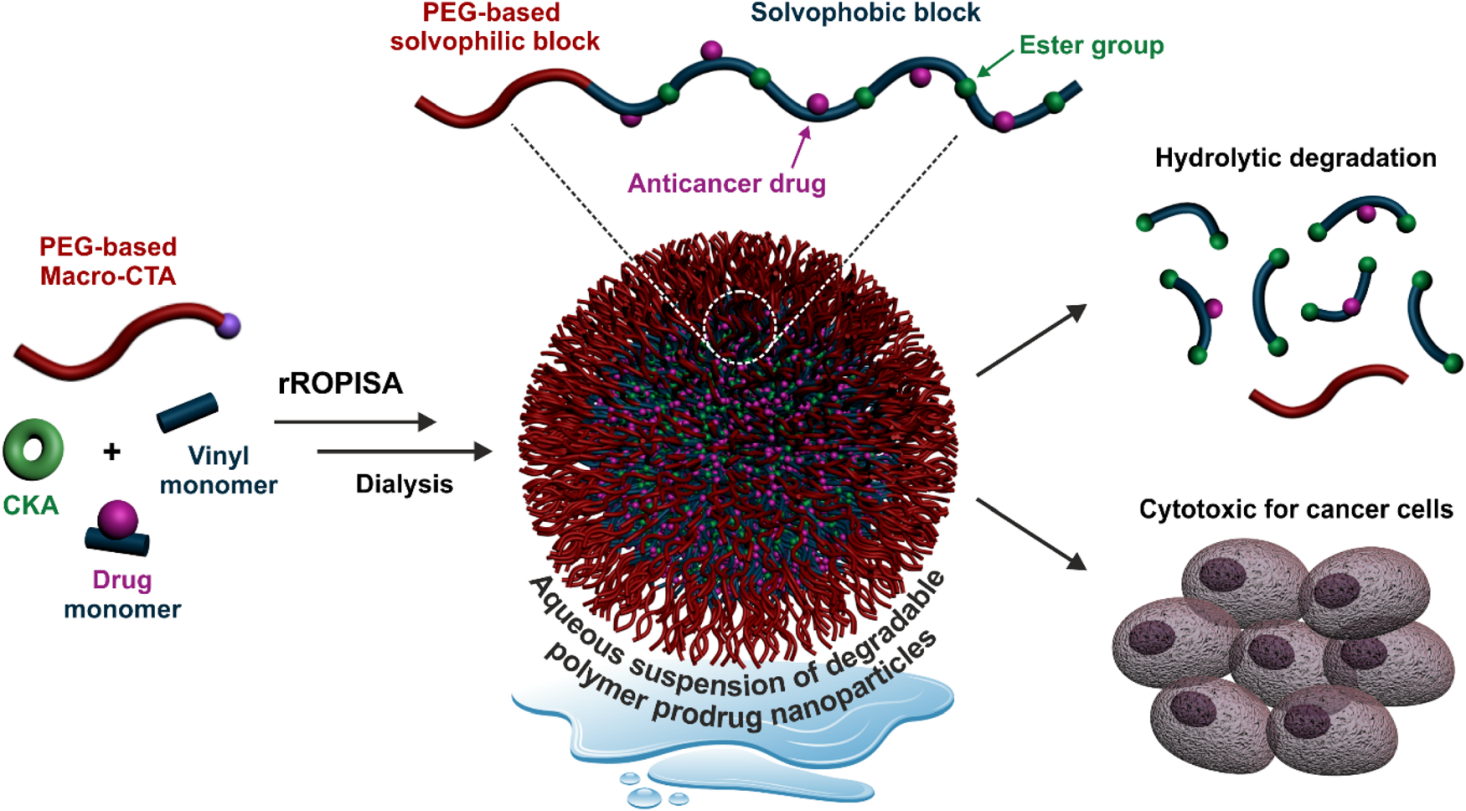
Synthesis of aqueous suspensions of core-degradable diblock copolymer prodrug nanoparticles by radical ring-opening polymerization-induced self-assembly (rROPISA) from cyclic ketene acetals (CKAs) and drug-bearing monomers.

Herein, by selecting appropriate experimental conditions, we report the synthesis of aqueous suspensions of degradable polymer prodrug nanoparticles by rROPISA (Fig. 1), by performing RAFT-mediated chain extension of a biocompatible solvophilic block with a combination of vinyl monomers, CKAs and drug-monomers. This allowed for the preparation of stable and narrowly dispersed polymer prodrug nanoparticles featuring adjustable drug loading, hydrolytic degradation and significant cytotoxicity against cancer cells. The versatility of this process was illustrated by its application to degradable polymer prodrug nanoparticles based on two different anticancer drugs, gemcitabine (Gem) and paclitaxel (Ptx), which are both widely used clinically against different types of cancers, under the names Gemzar and Taxol, respectively. Moreover, the polymer prodrug nanoparticles were readily fluorescently labeled for imaging purposes by insertion of fluorescent moieties in the copolymer backbone during the rROPISA process.

## Materials and methods

### Materials

Methacrylic acid, 4-dimethylaminopyridine, 1-hydroxybenzotriazole, 1-(3-dimethylaminopropyl) 3-ethylcarbodiimide hydrochloride, mono-2-methacyloyloxy ethyl succinate, lauryl methacrylate (LMA, 96%) and oligo(ethylene glycol) methyl ether methacrylate (OEGMA, *M*_*n*_ = 300 g mol^−1^) were purchased from Sigma-Aldrich. LMA and OEGMA were passed through basic alumina before use. 2,2′-Azobis(2-methylpropionitrile) (AIBN, 98%), 4-cyano-4-[(dodecylsulfanylthiocarbonyl)sulfanyl]pentanoic acid (CDSPA, 97%), anhydrous *N*,*N*′-dimethylformamide (DMF), dichloromethane (DCM) and methanol (MeOH) were purchased from Sigma-Aldrich and used as received. *Tert*-butyl peroxy-2-ethylhexanoate (Trigonox 21S or T21s) was supplied by AkzoNobel. Methacryloxyethyl thiocarbamoyl rhodamine B (RhoMA) was purchased from Polysciences and used as received. Deuterated chloroform (CDCl_3_), tetrahydrofuran (THF) and dimethyl sulfoxide (DMSO) were obtained from Eurisotop. 2-Methylene-4-phenyl-1,3-dioxolane (MPDL) and 5,6-benzo-2-methylene-1,3-dioxepane (BMDO) were prepared from the cyclic bromoacetal intermediate as described elsewhere.^44^ All other solvents were purchased from Carlo-Erba and used as received. [3-(4,5-Dimethylthiazol-2-yl)-2,5-diphenyl tetrazolium bromide] (MTT) was purchased from Sigma-Aldrich and used as received. Gemcitabine·HCl (Gem·HCl, >98%) was purchased from Carbosynth Limited (UK). Paclitaxel (Ptx, >95%) was purchased from Sequoia Research Products Limited (UK).

### Analytical method

#### Nuclear magnetic resonance (NMR) spectroscopy

Samples were solubilized in deuterated chloroform (CDCl_3_), dimethyl sulfoxide (*d*_6_-DMSO) or tetrahydrofuran (TDF), and placed in 5 mm diameter tubes for ^1^H-NMR spectroscopy at 25 °C on a Bruker Avance 300 spectrometer at 300 MHz. The chemical shift scale was calibrated based on the internal solvent signal (CDCl_3_: *δ* = 7.26 ppm; *d*_6_-DMSO: *δ* = 2.50 ppm; TDF: *δ* = 1.72 and 3.58 ppm).

#### Size exclusion chromatography (SEC)

SEC measurements were performed at 35 °C with two columns from Polymer Laboratories (PL-gel MIXED-D; 300 × 7.5 mm; bead diameter, 5 μm; linear part, 400–400 000 g mol^−1^) and a differential refractive index detector (Spectrasystem RI-150 from Thermo Electron Corp.). Chloroform was used as the eluent at a flow rate of 1 mL min^−1^ and toluene (0.5% v/v) was added as a flow-rate marker. A conventional calibration curve was based on poly(methyl methacrylate) (PMMA) standards (peak molar masses, *M*_p_ = 625–625 500 g mol^−1^) from Polymer Laboratories. This technique allowed for the determination of *M*_*n*_ (number-average molar mass), *M*_w_ (weight-average molar mass), and *M*_w_/*M*_*n*_ (dispersity, *Đ*).

#### Dynamic light scattering (DLS)

Nanoparticle diameters, reported in number (*D*_*n*_), volume (*D*_v_) and intensity (*D*_*z*_), as well as particle size distribution (PSD) were measured by dynamic light scattering (DLS) with a Nano ZS from Malvern (173° scattering angle) at a temperature of 25 °C, using the set parameters for each dispersing medium.

#### Transmission electron microscopy (TEM)

Grids were glow-discharged before use. 5 μL of nanoparticle suspensions were deposited for 30 s on copper grids covered with formvar–carbon film. The excess solution was blotted off using a piece of filter paper. Samples were then stained using uranyl acetate (2%, w/v) for 1 min at room temperature. Then the excess solution was removed using a piece of filter paper. The grids were then analyzed using a JEOL JEM-1400 operating at 80 kV. Images were acquired using an Orius camera (Gatan Inc, USA). The nanoparticles were analyzed by defining the number-average diameter (*d*_*n*_), the weight-average diameter (*d*_w_), the *z*-average diameter (*d*_*z*_) and the polydispersity index (PDI) using the following equations:
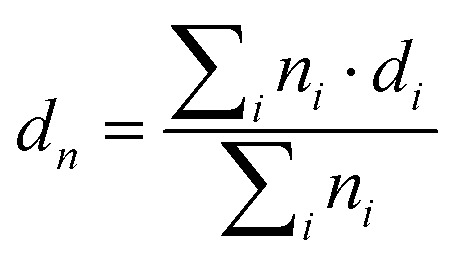

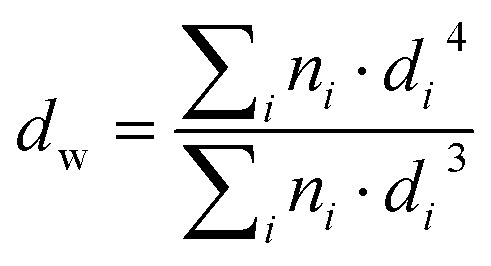

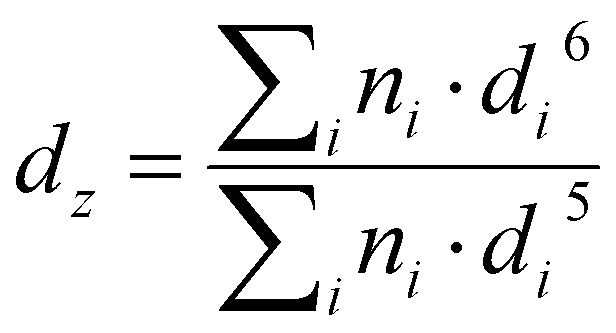
PDI = *d*_w_/*d*_*n*_

### Synthetic procedures

#### Synthesis of paclitaxel methacrylate (PtxMA)

In a 50-mL one-neck round-bottom flask equipped with a magnetic stirrer, Ptx (341.6 mg, 0.4 mmol) and triethylamine (141.4 mg, 1.4 mmol) in anhydrous DCM (20 mL) were added to a solution of methacrylic acid (120.4 mg, 1.4 mmol), 4-dimethylaminopyridine (85.4 mg, 0.70 mmol) and 1-(3-dimethylaminopropyl) 3-ethylcarbodiimide hydrochloride (217.0 mg, 1.4 mmol) in DCM (10 mL). The reaction mixture was purged with argon and stirred overnight at room temperature under an argon atmosphere. The reaction mixture was then washed with 1 M HCl (3 × 10 mL) and distilled water (3 × 10 mL). The aqueous phase was collected and washed with DCM (3 × 10 mL). The combined organic phases were washed with brine (3 × 10 mL), dried using magnesium sulfate (MgSO_4_) and the solvent was removed under reduced pressure. The crude residue was purified by column chromatography with ethyl acetate : *n*-hexane (2 : 1; v/v) as the mobile phase (*R*_f_ = 0.64), giving PtxMA as a white powder (348 mg; 63% yield). LC/MS: 922.4 g mol^−1^, 100%. ^1^H-NMR (300 MHz, CDCl_3_): *δ* (ppm) 7.30–8.15 (m, 15H, aromatic protons), 6.94 (d, 1H, –CO–NH–), 6.30 (s, 1H, –COO–CH), 6.25 (t, 1H, –NH–C**H**-Ar), 6.15 (s, 1H, –CH_3_–C=C**H**_2_), 5.96 (dd, 1H, –COO–C**H**–CH_2_), 5.64–5.74 (m, 2H, –O–CH, –CH_3_–C=C**H**_2_), 5.51 (d, 1H, –COO–CH–O–), 5.00 (d, 1H, –CH_2_–C**H**–O), 4.45 (t, 1H, –CH_2_–C**H**–OH), 4.18–4.34 (dd, 2H, –C**H**_2_–O–CH), 3.80 (d, 1H, –C**H**–CH–OOC), 2.51 (m, 1H, –CH-C**H**_2_–CH), 2.43 (s, 3H, –COO-CH_3_), 2.32–2.65 (m, 2H, –CH–C**H**_2_–CH–O), 2.22 (s, 3H, –COO–CH_3_), 2.10–2.20 (m, 1H, –CH_2_–CH–O**H**), 1.96 (s, 3H, CH_3_–C=C), 1.94 (s, 3H, –C**H**_3_–C=CH_2_), 1.84–1.90 (m, 1H, –C–OH), 1.65–1.75 (m, 4H, –C–CH_3_, –CH–C**H**_2_–CH–), 1.23 (s, 3H, –C–CH_3_), 1.13 (s, 3H, –C–CH_3_).

#### Synthesis of gemcitabine methacrylate (GemMA)

In a 50-mL one-neck round bottom flask equipped with a magnetic stirrer, Gem·HCl (651 mg, 2.17 mmol), 1-hydroxybenzotriazole (333 mg, 2.17 mmol), 1-(3-dimethylaminopropyl) 3-ethylcarbodiimide hydrochloride (417 mg, 2.17 mmol) and mono-2-methacyloyloxy ethyl succinate (500 mg, 2.17 mmol) were dissolved in dry DMF (26 mL) and pyridine (2 mL). The reaction mixture was purged with argon and stirred overnight at room temperature under an argon atmosphere. The reaction mixture was then concentrated under vacuum and ethyl acetate was added (50 mL). The organic phase was washed with 10% NaHCO_3_ aqueous solution (3 × 50 mL) and dried over MgSO_4_. The solution was concentrated under reduced pressure and the product was purified *via* column chromatography with DCM : MeOH (15 : 1; v/v) as the mobile phase (*R*_f_ = 0.26), giving GemMA as a white powder (413 mg, 40% yield). LC/MS: 476 g mol^−1^, 100%. ^1^H-NMR (300 MHz, *d*_6_-DMSO): *δ* (ppm) 1.86 (s, 3H, –CH_3_), 2.61 (m, 2H, –O–C(O)–CH_2_–), 2.80 (m, 2H, –CH_2_–C(O)–NH–), 3.66–3.79 (m, 2H, –CH_2_–OH, 2H), 3.90 (m, 1H, –CH–O–), 4.20 (m, 1H, –CH(OH)–), 4.29 (s, 4H, –O–CH_2_–, CH_2_–O–), 5.27 (t, 1H, CH_2_–OH), 5.66 (quint, 1H, HCH=C(CH_3_)–), 6.02 (quint, 1H, HCH=C(CH_3_)–), 6.19 (t, 1H, –C–CH=CH–N–), 6.28 (d, 1H, –CH–OH), 7.21 (d, 1H, –N–CH–O), 8.22 (d, 1H, C–CH=CH–N–), 11.05 (s, 1H, –C(O)–NH–); ^13^C-NMR (300 MHz, *d*_6_-DMSO): *δ* (ppm) 18.4 (–CH_3_), 28.7 (–O–C(O)–CH_2_–), 31.6 (–CH_2_–C(O)–NH–), 59.6 (–CH_2_–OH), 62.3 (–O–CH_2_–CH_2_–O–), 63.0 (–O–CH_2_–CH_2_–O–), 68.8 (–CH–OH), 81.4 (–CH–CH_2_–OH), 95.6 (–CH–CH=CH–N–), 110.3 (–O–C(CF_2_)–N), 116.2 (–C–F), 126.5 (CH_2_=C(CH_3_)–), 136.0 (–C(CH_3_)=CH_2_), 145.2 (–CH=CH–N–), 154.7 (–N–C(O)–N), 163.3 (–NH–C=N–), 166.7 (–C(O)–O–), 172.6 (–CH_2_–O–C(O)–CH_2_), 173.1 (–C(O)–NH–).

#### Synthesis of the poly[oligo(ethylene glycol) methyl ether methacrylate] (POEGMA) macro-chain transfer agent (CTA)

OEGMA (3.680 g, 0.012 mol), AIBN (9.6 mg, 5.93 × 10^−2^ mmol), CDSPA (0.097 g, 2.41 × 10^−1^ mmol, CDSPA/AIBN molar ratio = 4.0) and acetonitrile (25 mL) were poured into a 50-mL round bottom flask along with a fitted rubber septum and a magnetic stirring bar. The solution was degassed for 20 min by argon bubbling and then immersed in a preheated oil bath at 70 °C for 5 h. The reaction was stopped by placing the reaction vessel in ice. Acetonitrile was then removed under reduced pressure and the polymer was precipitated in a large excess of a cold diethyl ether/petroleum spirit mixture (1 : 1; v/v). The purified polymer was dried under high vacuum until constant weight. Monomer conversion was determined by ^1^H-NMR spectroscopy by integrating the two oxymethylene proton signals of OEGMA and POEGMA at 4.3 and 4.1 ppm, respectively. *DP*_*n*,NMR_ was determined by ^1^H-NMR spectroscopy by integrating the two oxymethylene proton signals of POEGMA at 4.1 ppm and the eighteen proton signals of the C_12_ alkyl chains of CDSPA at 1.2–1.4 ppm. SEC analysis was carried out on the purified polymer: POEGMA_28_ (*M*_*n*_ = 8900, *Đ* = 1.10).

The synthesis of poly[oligo(ethylene glycol) methyl ether methacrylate-*co*-methacryloxyethyl thiocarbamoyl rhodamine B] P(OEGMA-*co*-RhoMA) was carried out following a similar procedure with *f*_RhoMA_ = 0.1 mol%: OEGMA (3.680 g, 0.012 mol), AIBN (9.6 mg, 5.93 × 10^−2^ mmol), CDSPA (0.097 g, 2.41 × 10^−1^ mmol, CDSPA/AIBN molar ratio = 4.0), methacryloxyethyl thiocarbamoyl rhodamine B (8.2 mg, 1.2 × 10^−2^ mmol) and acetonitrile (25 mL). To ensure the compete removal of the free RhoMA monomer after copolymerization, the copolymer was precipitated in a large excess of a cold diethyl ether/petroleum spirit mixture (1 : 1; v/v) and solubilized in a minimum amount of MeOH followed by dialysis with a RC dialysis membrane (MWCO = 3.5 kDa) against MeOH for five days. SEC analysis was carried out on the purified polymer: P(OEGMA_24_-*co*-RhoMA) (*M*_*n*_ = 8900, *Đ* = 1.08).

#### Synthesis of degradable block copolymer nanoparticles

##### Synthesis of poly[oligo(ethylene glycol)methyl ether methacrylate]-*b*-poly[(lauryl methacrylate-*co*-5,6-benzo-2-methylene-1,3-dioxepane-*co*-paclitaxel methacrylate)] (POEGMA-*b*-P(LMA-*co*-BMDO-*co*-PtxMA))

A typical synthesis of POEGMA_28_-*b*-P(LMA_150_-*co*-BMDO) with [PtxMA] : [LMA] : [BMDO] = 0 : 1 : 2 (C0), was performed by reversible addition–fragmentation chain-transfer (RAFT) dispersion copolymerization at 20 wt% solids, as follows. In a 7-mL vial fitted with a rubber septum and a magnetic stirring bar, a mixture of LMA (0.203 g, 8.00 × 10^−4^ mol, *DP*_*n*,th_ = 150), BMDO (0.256 g, 1.58 × 10^−3^ mol), T21s initiator (0.2 mg, 9.26 × 10^−4^ mmol, dissolved at 0.1% w/v in DMF) and POEGMA_28_ macro-CTA (0.048 g, 5.33 × 10^−3^ mmol; macro-CTA/initiator molar ratio = 5.0) in anhydrous DMF (2 g, 2.1 mL) was degassed by argon bubbling for at least 15 min and then heated in a preheated oil bath at 90 °C for 24 h. The reaction was stopped by placing the reaction vessel in an ice bath. LMA conversion was determined by ^1^H-NMR spectroscopy by integrating the two oxymethylene protons of LMA at 5.5 and 6.0 ppm with PLMA protons at 3.8 ppm. *M*_*n*_ and *Đ* were determined by SEC. The nanoparticles were poured into a pre-wetted dialysis bag (MWCO 3500, RC membrane) and dialyzed against acetone or DMF (for the fluorescent nanoparticles). The dialysate was changed twice a day for three days. After dialysis, the suspension of nanoparticles was transferred to another dialysis bag (MWCO = 3500, RC membrane) and dialyzed against Mili-Q water. Water was changed twice a day for three days. The nanoparticle colloidal characteristics (*D*_*z*_ and PSD) were obtained by DLS.

The same procedure was carried out with variable amounts of PtxMA and BMDO, as follows: with [PtxMA] : [LMA] : [BMDO] = 0.1 : 0.9 : 0 (PT1) [LMA (0.311 g, 1.23 × 10^−3^ mol, *DP*_*n*,th_ = 150), T21s initiator (0.4 mg, 1.85 × 10^−3^ mmol, dissolved at 0.1% w/v in DMF), PtxMA (0.126 g, 1.37 × 10^−1^ mmol) and POEGMA_28_ macro-CTA (0.081 g, 9.00 × 10^−3^ mmol; macro-CTA/initiator molar ratio = 5.0) in anhydrous DMF (2 g, 2.1 mL)]; with [PtxMA] : [LMA] : [BMDO] = 0.05 : 0.95 : 2 (PT2) [LMA (0.192 g, 7.54 × 10^−4^ mol, *DP*_*n*,th_ = 150), BMDO (0.247 g, 1.52 × 10^−3^ mol), T21s initiator (0.2 mg, 9.26 × 10^−4^ mmol, dissolved at 0.1% w/v in DMF), PtxMA (0.033 g, 3.58 × 10^−2^ mmol) and POEGMA_28_ macro-CTA (0.038 g, 5.05 × 10^−3^ mmol; macro-CTA/initiator molar ratio = 5.0) in anhydrous DMF (2 g, 2.1 mL)]; with [PtxMA] : [LMA] : [BMDO] = 0.1 : 0.9 : 2 (PT3) [LMA (0.164 g, 6.46 × 10^−4^ mol, *DP*_*n*,th_ = 150), BMDO (0.236 g, 1.46 × 10^−3^ mol), T21s initiator (0.2 mg, 9.26 × 10^−4^ mmol, dissolved at 0.1% w/v in DMF), PtxMA (0.064 g, 6.94 × 10^−2^ mmol) and POEGMA_28_ macro-CTA (0.036 g, 4.74 × 10^−3^ mmol; macro-CTA/initiator molar ratio = 5.0) in anhydrous DMF (2 g, 2.1 mL)]; with [PtxMA] : [LMA] : [BMDO] = 0.2 : 0.8 : 2 (PT4) [LMA (0.141 g, 5.55 × 10^−4^ mol, *DP*_*n*,th_ = 150), BMDO (0.214 g, 1.32 × 10^−3^ mol), T21s initiator (0.2 mg, 9.26 × 10^−4^ mmol, dissolved at 0.1% w/v in DMF), PtxMA (0.122 g, 1.32 × 10^−1^ mmol) and POEGMA_28_ macro-CTA (0.035 g, 4.60 × 10^−3^ mmol; macro-CTA/initiator molar ratio = 5.0) in anhydrous DMF (2 g, 2.1 mL)] and with [PtxMA] : [LMA] : [BMDO] = 0.3 : 0.7 : 2 (PT5) [LMA (0.116 g, 4.57 × 10^−4^ mol, *DP*_*n*,th_ = 150), BMDO (0.160 g, 1.74 × 10^−4^ mol), T21s initiator (0.2 mg, 9.26 × 10^−4^ mmol, dissolved at 0.1% w/v in DMF), PtxMA (0.160 g, 1.74 × 10^−1^ mmol) and POEGMA_28_ macro-CTA (0.032 g, 4.22 × 10^−3^ mmol; macro-CTA/initiator molar ratio = 5.0) in anhydrous DMF (2 g, 2.1 mL)].

##### Synthesis of the poly[oligo(ethylene glycol)methyl ether methacrylate]-*b*-poly[(lauryl methacrylate-*co*-2-methylene-4-phenyl-1,3-dioxolane-*co*-paclitaxel methacrylate)] (POEGMA-*b*-P(LMA-*co*-MPDL-*co*-PtxMA)) copolymer

The same procedure was carried out in the presence of PtxMA and MPDL, with [PtxMA] : [LMA] : [MPDL] = 0.2 : 0.8 : 2 (PT6) [LMA (0.164 g, 6.46 × 10^−4^ mol, *DP*_*n*,th_ = 140), MPDL (0.215 g, 1.33 × 10^−3^ mol), T21 s initiator (0.2 mg, 9.26 × 10^−4^ mmol, dissolved at 0.1% w/v in DMF), PtxMA (0.064 g, 6.94 × 10^−2^ mmol) and POEGMA_28_ macro-CTA (0.036 g, 4.74 × 10^−3^ mmol; macro-CTA/initiator molar ratio = 5.0) in anhydrous DMF (2 g, 2.1 mL)].

##### Synthesis of the poly[oligo(ethylene glycol) methyl ether methacrylate-*co*-methacryloxyethyl thiocarbamoyl rhodamine B]-*b*-poly[(lauryl methacrylate-*co*-5,6-benzo-2-methylene-1,3-dioxepane-*co*-paclitaxel methacrylate)] (P(OEGMA-*co*-RhoMA)-*b*-P(LMA-*co*-BMDO-*co*-PtxMA)) copolymer

The same procedure was carried out using P(OEGMA_24_-*co*-RhoMA) macro-CTA in the presence of PtxMA and BMDO, with [PtxMA] : [LMA] : [BMDO] = 0.05 : 0.95 : 2 (PT2*) [LMA (0.181 g, 7.13 × 10^−4^ mol, *DP*_*n*,th_ = 140), PtxMA (0.039 g, 4.23 × 10^−5^ mol), T21s initiator (0.2 mg, 9.26 × 10^−4^ mmol, dissolved at 0.1% w/v in DMF), and P(OEGMA_24_-*co*-RhoMA) macro-CTA (0.044 g, 4.98 × 10^−3^ mmol; macro-CTA/initiator molar ratio = 5.0) in anhydrous DMF (2 g, 2.1 mL)]. The same procedure was carried out without PtxMA (C0-Rho) [LMA (0.200 g, 7.87 × 10^−4^ mol, *DP*_*n*,th_ = 150), BMDO (0.258 g, 1.57 × 10^−3^ mol), T21s initiator (0.2 mg, 9.26 × 10^−4^ mmol, dissolved at 0.1% w/v in DMF), P(OEGMA_24_-*co*-RhoMA) macro-CTA (0.047 g, 5.27 × 10^−3^ mmol; macro-CTA/initiator molar ratio = 5.0) in anhydrous DMF (2 g, 2.1 mL)].

##### Synthesis of poly[oligo(ethylene glycol)methyl ether methacrylate]-*b*-poly[(lauryl methacrylate-*co*-5,6-benzo-2-methylene-1,3-dioxepane-*co*-gemcitabine methacrylate)] (POEGMA-*b*-P(LMA-*co*-BMDO-*co*-GemMA))

The same procedure was carried out in the presence of variable amounts of GemMA and BMDO, with [GemMA] : [LMA] : [BMDO] = 0.1 : 0.9 : 0 (G1) [LMA (0.35 g, 1.38 × 10^−3^ mol, *DP*_*n*,th_ = 140), GemMA (0.071 g, 1.49 × 10^−4^ mol), T21s initiator (0.4 mg, 1.85 × 10^−3^ mmol, dissolved at 0.1% w/v in DMF), and POEGMA_28_ macro-CTA (0.089 g, 9.90 × 10^−3^ mmol; macro-CTA/initiator molar ratio = 5.0) in anhydrous DMF (2 g, 2.1 mL)]; with [GemMA] : [LMA] : [BMDO] = 0.1 : 0.9 : 2 (G2) [LMA (0.176 g, 6.93 × 10^−4^ mol, *DP*_*n*,th_ = 140), BMDO (0.249 g, 1.54 × 10^−3^ mol), T21s initiator (0.2 mg, 9.26 × 10^−4^ mmol, dissolved at 0.1% w/v in DMF), GemMA (0.037 g, 7.77 × 10^−2^ mmol) and POEGMA_28_ macro-CTA (0.039 g, 5.13 × 10^−3^ mmol; macro-CTA/initiator molar ratio = 5.0) in anhydrous DMF (2 g, 2.1 mL)] and with [GemMA] : [LMA] : [BMDO] = 0.2 : 0.8 : 2 (G3) [LMA (0.153 g, 6.02 × 10^−4^ mol, *DP*_*n*,th_ = 150), BMDO (0.239 g, 1.48 × 10^−3^ mol), T21s initiator (0.2 mg, 9.26 × 10^−4^ mmol, dissolved at 0.1% w/v in DMF), GemMA (0.073 g, 1.53 × 10^−1^ mmol) and POEGMA_28_ macro-CTA (0.037 g, 4.07 × 10^−3^ mmol; macro-CTA/initiator molar ratio = 5.0) in anhydrous DMF (2 g, 2.1 mL)].

##### Synthesis of poly[oligo(ethylene glycol)methyl ether methacrylate-*co*-methacryloxyethyl thiocarbamoyl rhodamine B]-*b*-poly[(lauryl methacrylate-*co*-5,6-benzo-2-methylene-1,3-dioxepane-*co*-gemcitabine methacrylate)] (P(OEGMA-*co*-RhoMA)-*b*-P(LMA-*co*-BMDO-*co*-GemMA))

The same procedure was carried out using P(OEGMA_24_-*co*-RhoMA) macro-CTA in the presence of GemMA and BMDO, with [GemMA] : [LMA] : [BMDO] = 0.1 : 0.9 : 2 (G2*) [LMA (0.173 g, 6.81 × 10^−4^ mol, *DP*_*n*,th_ = 135), BMDO (0.245 g, 1.51 × 10^−3^ mol), GemMA (0.036 g, 7.57 × 10^−5^ mol), T21s initiator (0.2 mg, 9.26 × 10^−4^ mmol, dissolved at 0.1% w/v in DMF), P(OEGMA_24_-*co*-RhoMA) macro-CTA (0.045 g, 5.05 × 10^−3^ mmol; macro-CTA/initiator molar ratio = 5.0) in anhydrous DMF (2 g, 2.1 mL)].

### Degradation procedures

#### Copolymer degradation

In a 5-mL vial, 10 mg of purified copolymer was dissolved in 0.5 mL of THF, followed by addition of 0.5 mL of potassium hydroxide solution (KOH, 5 wt%) in MeOH. The cloudy mixture was stirred at room temperature. Samples were collected and dried overnight under vacuum, followed by the addition of 2 mL of chloroform and three washes with HCl (1 mL, 1 mol L^−1^). After extraction, the organic phase was filtered with a 0.2 μm PTFE filter and dried under reduced pressure. The degradation products were analyzed by SEC.

#### Nanoparticle degradation

In a 40-mL vial, an aqueous suspension of copolymer nanoparticles was mixed with an equal volume of aqueous potassium hydroxide solution (KOH, 5 wt%). The vial was placed in an orbital shaker (IKA KS4000i control, 37 °C, 90 rpm) and samples were withdrawn at different intervals, and then lyophilized. 2 mL of chloroform was added, followed by three washes with HCl (1 mL, 1 mol L^−1^). After extraction, the organic phase was filtered with a 0.2 μm PTFE filter and dried under reduced pressure. The degradation products were analyzed by SEC.

#### Biological evaluation

##### Cell culture

Lung adenocarcinoma (A549) cells were purchased from the American Type Culture Collection (ATCC) and maintained as recommended. Fetal Bovine Serum (FBS) was purchased from Gibco. Penicillin–streptomycin stabilized solution and Roswell Park Memorial Institute medium (RPMI)-1640 were purchased from Sigma-Aldrich and used as received.

##### Cell viability assay

In 96-well microtiter plates (TPP, Switzerland), A549 cells were seeded (5 × 10^4^ cells per mL) in 100 μL of growth medium and preincubated for 24 h in an incubator (37 °C and 5% CO_2_). Free Ptx was dissolved in DMSO, followed by appropriate dilutions in cell culture medium (10 mM in DMSO). After appropriate dilutions, 100 μL of aqueous copolymer nanoparticles in cell culture medium were added to the cells and incubated for 72 h. A MTT solution (5 mg mL^−1^) was prepared with phosphate buffered saline (PBS) and filtered with sterile filters (0.2 μm). At the end of the incubation period, 20 μL of MTT solution was added to each well. After 1 h of incubation, the medium was removed and 200 μL of dimethylsulfoxide (DMSO) was then added to each well to dissolve the formazan crystals. The absorbance was then measured using a microplate reader (LAB Systems Original Multiscan MS) at 570 nm. Cell viability was calculated as the absorbance ratio between treated and untreated control cells. All experiments were performed in triplicate to determine the means and SD.

#### Confocal microscopy

A549 cells (2 mL, 1.75 × 10^5^ cells per mL) were seeded in a 6-well plate containing a sterilized coverslip (0.17 mm thick, 25 mm diameter) and incubated overnight to reach ∼70% confluence. Then, 2 mL of fluorescent nanoparticles (PT2* and G2*) diluted in growth medium (1 mg mL^−1^) were added. The plate was incubated at 37 °C with 5% CO_2_. After 2 h or 4 h, the coverslip was placed in a microscopy cell chamber (Attofluor® cell Chamber) and 500 μL of calcein-AM (3 μM in PBS) was added. The coverslip was incubated for 5–10 min, followed by the removal of the calcein-AM mixture. Cells were washed three times with 1 mL PBS and refilled with the same volume of medium. The prepared coverslip was transferred into an Attofluor® cell chamber and imaged by confocal laser scanning microscopy (Zeiss). The green fluorescence emission was detected at *λ* = 505–550 nm and the red fluorescence emission was detected at *λ* = 585–700 nm under sequential mode. DIC (Differential Interferential Contrast) images were obtained simultaneously during the imaging process. The pinhole diameter was set at 1.0 Airy unit. Twelve-bit numerical images were acquired with LSM 510 software version 3.2 and the resulting images were analyzed using ImageJ software.

## Results and discussion

### General synthetic route

**Fig. 2:**
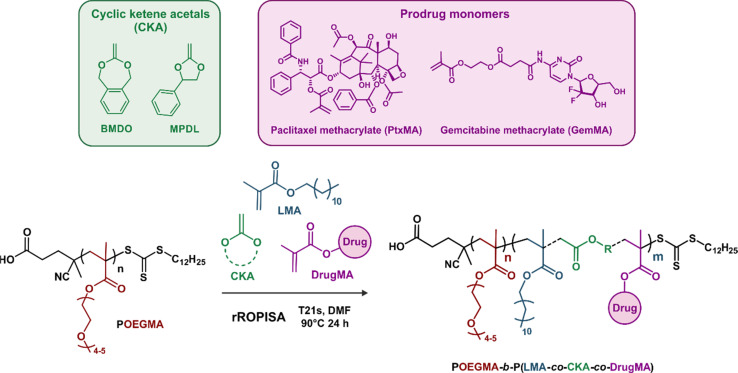
RAFT-mediated chain extension of the poly[oligo(ethylene glycol) methyl ether methacrylate] (POEGMA) macro-RAFT agent in DMF with lauryl methacrylate (LMA), cyclic ketene acetals (CKAs) and drug-bearing methacrylates (DrugMA) by rROPISA, followed by transfer of the resulting nanoparticles to water to produce aqueous suspensions of POEGMA-*b*-P(LMA-*co*-CKA-*co*-DrugMA) diblock copolymer prodrug nanoparticles.

To generate aqueous suspensions of degradable polymer prodrug nanoparticles by rROPISA, we carried out chain extension by RAFT polymerization of a poly[oligo(ethylene glycol) methyl ether methacrylate] (POEGMA) solvophilic block, to form a biocompatible nanoparticle shell. The nanoparticle degradable core, to which multiple drug molecules are covalently linked, was composed of lauryl methacrylate (LMA), variable amounts of CKAs (2-methylene-4-phenyl-1,3-dioxolane (MPDL) or 5,6-benzo-2-methylene-1,3-dioxepane (BMDO)) and anticancer drug-bearing methacrylate monomers (Fig. 2). To illustrate the versatility and robustness of the process, we investigated two different drug-bearing methacrylates: paclitaxel-methacrylate (PtxMA) and gemcitabine-methacrylate (GemMA).

### Paclitaxel-based polymer prodrug nanoparticles by rROPISA

Derivatization of Ptx was successfully carried out by esterification of its C-2′-hydroxyl group with methacrylic acid^45^ in the presence of EDC and DMAP. The disappearance of the C2′ hydroxyl group at 3.64 ppm and the appearance of vinylic protons at 5.7 and 6.2 ppm in the purified product, as shown in the ^1^H-NMR spectrum, together with LC/MS analysis, confirmed the successful formation of PtxMA with 63% yield (Fig. S1†).

To form the solvophilic block, a POEGMA_28_ (*M*_*n*,SEC_ = 8900 g mol^−1^, *Đ* = 1.10, Table S1†) macro-chain transfer agent was synthesized by RAFT polymerization of OEGMA in acetonitrile at 70 °C under AIBN initiation (Fig. S2†).

Its chain extension was first carried out at 20 wt% solids with LMA (targeted *DP*_*n*,PLMA_ = 150) and BMDO (*f*_BMDO,0_ = 0.66) in DMF at 90 °C for 24 h, in the presence of T21s as the initiator (C0, Table 1). ^1^H-NMR analysis of the resulting POEGMA-*b*-P(LMA-*co*-BMDO) diblock copolymers confirmed the formation of the expected structure and the successful insertion of BMDO in the solvophobic block (*F*_BMDO_ = 0.07), as supported by the presence of peaks *g* (aromatic ring of BMDO) and *j* (methylene protons characteristic of open BMDO) (Fig. S3†). A similar rROPISA was then carried out in the presence of PtxMA (*f*_PtxMA,0_ = 0.1), but without BMDO (PT1, Table 1). As expected, characteristic protons from Ptx were present in the ^1^H-NMR spectrum (see peaks *m*, *n* and *o* in Fig. S3†), confirming the successful synthesis of the POEGMA-*b*-P(LMA-*co*-PtxMA) diblock copolymer prodrug. The drug loading of Ptx was found to be 11 wt%, with *F*_PtxMA_ being lower than *f*_PtxMA,0_, likely because of the large steric hindrance of the Ptx moiety. Note the significantly higher LMA conversion for PT1 compared to C0 (89 *vs.* 57%), which can be explained by the unfavorable reactivity ratios generally observed between CKAs and methacrylates, resulting in slower copolymerization rates.^41,43^

**Table 1:** Macromolecular characteristics of POEGMA-*b*-P(LMA-*co*-CKA-*co*-PtxMA) diblock copolymers

Ref.	[PtxMA] : [LMA] : [CKA]^a^	Conv.^b^ (%)	*F*_CKA_ c	*F*_PtxMA_ c	DL_Ptx_^d^ (wt%)	*M*_*n*,SEC_ e (g mol^−1^)	*M*_w,SEC_ e (g mol^−1^)	*Đ* e	*M*_*n*,exp_ after degradation^e^ (g mol^−1^)	*M*_w,exp_ after degradation^e^ (g mol^−1^)	*M*_*n*_ decrease^f^ (%)	*M*_w_ decrease^f^ (%)
C0	0 : 1 : 2	57	0.07	0	0	22 100	38 600	1.72	5400	10 200	−76	−74
PT1	0.10 : 0.90 : 0	89	0	0.05	11	29 500	41 900	1.45	28 300	41 500	−4	−1
PT2	0.05 : 0.95 : 2	59	0.16	0.01	3	21 600	35 400	1.46	3000	5600	−86	−84
PT3	0.10 : 0.90 : 2	63	0.13	0.06	13	21 400	34 140	1.60	4800	7800	−78	−77
PT4	0.20 : 0.80 : 2	68	0.09	0.10	19	16 800	28 700	1.71	5000	11 600	−70	−60
PT5	0.30 : 0.70 : 2	67	0.11	0.21	33	20 300	35 300	1.58	2700	4900	−87	−86
PT6	0.20 : 0.80 : 2	62	0.26	0.10	20	17 800	30 100	1.74	2500	10 100	−86	−66

a BMDO was used for C0 and PT1–PT5 and MPDL was used for PT6.

b LMA conversion determined by ^1^H-NMR, by integrating the two oxymethylene protons of LMA (5.5 and 6.0 ppm) and PLMA (3.8 ppm).

c Molar fraction of CKA and PtxMA in the solvophobic block determined by ^1^H-NMR, by integrating the 5H of MPDL or 4H of BMDO (7.0–8.0 ppm), excluding the 13H from Ptx and the 2H of LMA units (3.8–4.0 ppm).

d Drug loading in Ptx determined by ^1^H-NMR, according to MW_Ptx_/*M*_*n*,copolymer_, with MW_Ptx_ = molecular weight of Ptx and *M*_*n*,copolymer_ = *M*_*n*_ of the polymer prodrug considered.

e Determined by SEC after purification by dialysis.

f *M*_*n*_ and *M*_w_ decrease after the degradation of copolymers under accelerated conditions, calculated according to (exp. *M*_*n*,SEC_ − initial *M*_*n*,SEC_)/initial *M*_*n*,SEC_.

To produce degradable polymer prodrug nanoparticles, rROPISA was then carried out in the presence of LMA, BMDO (*f*_BMDO,0_ = 0.66) and increasing amounts of PtxMA (*f*_PtxMA,0_ = 0.016–0.10), under the same experimental conditions (PT2–5, Table 1). Overall, relatively high conversions in LMA were achieved (59–68%), with no clear trend observed in terms of *M*_*n*_ or dispersity. Interestingly, by adjusting the initial stoichiometry, it was possible to finely tune the amount of Ptx in the copolymer as the drug loading linearly increased with *f*_PtxMA,0_ from 3 to 33 wt%, while *F*_BMDO_ ranged from 9 to 16 mol% with no clear trend (Table 1). Switching from BMDO to MPDL did not affect *F*_PtxMA_ but resulted in a higher *F*_CKA_ (PT6).

In terms of colloidal characteristics, the synthesis of POEGMA_28_-*b*-P(LMA-*co*-CKA-*co*-PtxMA) diblock copolymer prodrugs by rROPISA successfully produced nanoparticles in DMF. For instance, nanoparticles C0 and PT1–PT4 exhibited intensity-weighted mean diameter (*D*_*z*_) between 48 and 125 nm, and low particle size distributions (PSD) in the range of 0.02–0.14, as shown by DLS (Table S2†). However, increasing *f*_PtxMA,0_ to 0.1 (PT5) yielded very small nanoparticles (*D*_*z*_ = 17 nm) and a broad PSD (Fig. S4†), which could be explained by the high solubility of PtxMA in DMF, preventing efficient copolymer self-assembly. Another reason could be a potential crystallinity driven self-assembly, which would be disrupted by the high PtxMA content. Also, when switching from BMDO to MPDL (PT6) for a fixed PtxMA content (*f*_PtxMA,0_ = 0.067), bigger particles with a broader PSD were obtained.

**Fig. 3:**
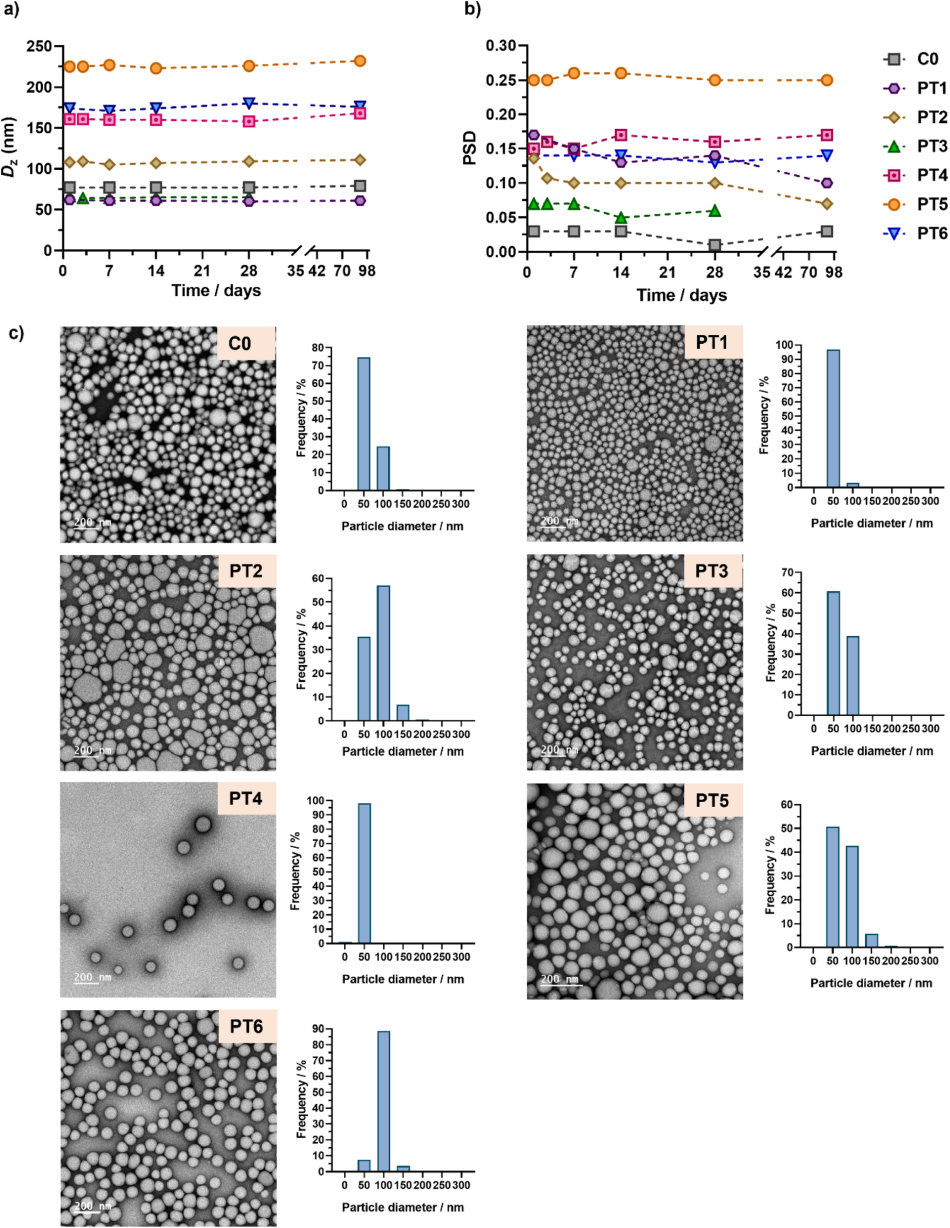
Evolution with time of (a) the intensity-weighted mean diameters (*D*_*z*_) and (b) the particle size distributions (PSD) of POEGMA_28_-*b*-P(LMA-*co*-CKA-*co*-PtxMA) nanoparticles C0 and PT1–PT6 determined by DLS after dialysis against water. (c) Representative TEM images and particle size distributions (*n* = 350–1000) of nanoparticles C0 and PT1–PT6 determined by TEM (*d*_*n*_, *d*_w_, *d*_*z*_, and polydispersity values are given in Table S3†).

Aqueous suspensions of POEGMA_28_-*b*-P(LMA-*co*-CKA-*co*-PtxMA) diblock copolymer prodrug nanoparticles were obtained after dialysis against water. This resulted in nanoparticles with no trace of residual unreacted monomer, as shown by the ^1^H-NMR of the dried sample after dialysis (Fig. S5†). The nanoparticles exhibited constant average diameters ranging from 77 to 225 nm, depending on the type of nanoparticle, and low polydispersities over a period of 1 to 3 months (Fig. 3a and b, Table S2†). Overall, their average diameters were rather well-preserved from DMF to water except, as expected, for PT5 which contained the highest amount of PtxMA units (Fig. S4†). Interestingly, both the average diameter and the particle size distribution seem to increase with increasing *F*_Ptx_, while the effect of *F*_CKA_ appears less obvious.

TEM images of each sample showed spherical morphologies regardless of the presence of CKA or PtxMA monomer units (Fig. 3c). In addition, the average sizes and size distributions of the nanoparticles were in good agreement with DLS measurements (Fig. 3d, Table S3†).

Degradation of the copolymers (obtained from the nanoparticle dry extracts) was carried out under accelerated conditions in THF with 5 wt% KOH (Table 1 and Fig. S6†). As expected, due to the absence of BMDO units, PT1 showed no decrease in *M*_*n*_, in contrast to C0, which exhibited a 76% decrease in *M*_*n*_ and whose SEC traces approached those of the starting macro-CTA (Fig. S6†), confirming the presence of ester groups in the copolymer chain (*F*_BMDO_ = 0.07). Degradation of copolymers PT2–PT5 under the same conditions yielded very similar results with a significant decrease in *M*_*n*_ in the 70–87% range, despite different *F*_BMDO_ values, supporting the successful synthesis of degradable polymer prodrugs. Using MPDL instead of BMDO under the same experimental conditions as PT4 (see PT6, Table 1) resulted in a very similar outcome (except for *F*_MPDL_, which was significantly higher than *F*_BMDO_), showing that the synthetic route is also applicable to other CKAs. Degradation of copolymer PT6 under accelerated conditions resulted in a smaller decrease in *M*_*n*_, which can be explained by the higher amount of close MPDL units compared to BMDO-based copolymers.^43^

**Fig. 4:**
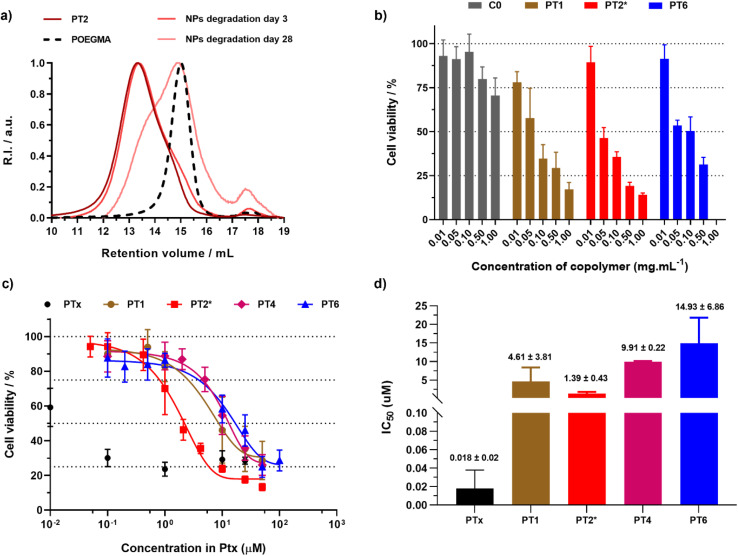
(a) SEC chromatograms after degradation of copolymers and nanoparticles PT2 under accelerated conditions. The dashed line represents the SEC traces of the corresponding POEGMA_28_ macro-CTA. (b) Cell viability (MTT assay) expressed in copolymer mass concentration after incubation of A549 cells with increasing concentrations of nanoparticles C0, PT1, PT2* and PT6 for 72 h. Results were expressed as the percentage of absorption of treated cells ± SD in comparison with untreated cells (control). (c) Cell viability (MTT assay) expressed in Ptx molar concentration and (d) corresponding IC_50_ values after incubation of A549 cells with free Ptx and nanoparticles PT1, PT2*, PT4 and PT6 for 72 h. Results were expressed as the percentage of absorption of treated cells ± SD in comparison with untreated cells (control). A One way ANOVA and an unpaired *t*-test were performed on PRISM 8.0.2 software. PT2*vs.PT1*p* value = 0.3262 (ns), *vs.*PT4*p* value < 0.0001 (****), *vs.*PT6*p* value = 0.0583 (ns).

The direct degradation of nanoparticles was first assessed under accelerated conditions (2.5 wt% KOH; pH = 14). Degradation of nanoparticles C0 (*F*_BMDO_ = 0.07) resulted in a clear shift of the SEC trace towards a lower *M*_*n*_ value, accounting for a 44% decrease in *M*_*n*_ after 3 days (Fig. S7†). It is interesting to note that, probably due to the high hydrophobicity and steric hindrance of Ptx, making access to the ester bond more difficult, the degradation of nanoparticles PT2 (*F*_BMDO_ = 0.16, *F*_Ptx_ = 0.01) was much slower than that of C0, showing only a 19% decrease in *M*_*n*_ after 3 days, but as high as 67% after 28 days (Fig. 4a).

It should be noted that degradation under accelerated conditions does not reflect *in vivo* conditions and only serves to probe the presence of ester groups in the copolymer backbone. However, similar polymethacrylates obtained by rROP have been shown to degrade in the long run (ranging from several months up to a year) under physiological conditions (*i.e.*, PBS, pH 7.4, 37 °C).^46^ We therefore expect similar behavior with these polymer prodrugs.

The anticancer cytotoxicity of the nanoparticles was then evaluated using cell viability assays on A549 lung cancer cells. As expected, Ptx-free nanoparticles C0 showed no toxicity (>70% cell viability) up to at least 1 mg mL^−1^ (Fig. S8†), demonstrating the good biocompatibility of the empty nanoparticles and of their building blocks. In contrast, all the polymer prodrug nanoparticles tested (PT1 and PT2* which are fluorescently labeled, PT4 and PT6) reached the IC_50_ value as early as 0.1 mg mL^−1^ (Fig. 4b), which suggested an efficient release of Ptx from the nanoparticles and a significant cytotoxic effect. The cytotoxicity results were also expressed in terms of the dose of Ptx (Fig. 4c and d). While free Ptx exhibited an IC_50_ value of 18 nM, in agreement with the literature,^47^ all polymer prodrug nanoparticles showed higher IC_50_ values, ranging from 1.4 to 14.9 μM. These results are in line with the prodrug concept, requiring cleavage of the drug-polymer linker before the active drug is released and can exert its cytotoxic effect. Since we have used a fairly short ester linker between Ptx (bulky and very hydrophobic) and the polymer backbone (also hydrophobic), even lower IC_50_ values could be reached by using a more hydrophilic, solvated linker thanks to the versatility of this synthetic approach.^47–49^ Importantly, nanoparticles PT2* showed greater cytotoxicity than nanoparticles PT1, demonstrating that synthesizing degradable polymer prodrug nanoparticles by rROPISA did not impair their cytotoxic effect, which is a significant improvement compared to the previous work in the field.^43^ Interestingly, nanoparticles PT1, PT4 and PT6 exhibited fairly similar IC_50_ values, suggesting that *F*_CKA_ or *F*_ptx_ do not contribute significantly to nanoparticle cytotoxicity, and that there is no significant impact of using BMDO rather than MPDL.

### Gemcitabine-based polymer prodrug nanoparticles by rROPISA

A similar synthetic strategy was applied to Gem to demonstrate the versatility and robustness of our degradable polymer prodrug nanoparticle design. Functionalization of Gem was carried out by amidation of mono-2-methacryloyloxy ethyl succinate methacrylate using carbodiimide chemistry to produce GemMA.^49,50^ Its successful preparation, with an overall yield of 40%, was confirmed by the appearance of vinylic (5.61 ppm) and methyl (1.8 ppm) protons, along with those from the ethyl succinate moiety (2.5–2.7 and 4.3 ppm) in the ^1^H-NMR spectrum (Fig. S9†). In addition, the amide carbon bond was observed by ^13^C-NMR and LC/MS analysis confirmed the successful conjugation.

rROPISA was carried out using the POEGMA_28_ macro-CTA at 20 wt% solids with LMA (targeted *DP*_*n*,PLMA_ = 150) and GemMA (*f*_GemMA,0_ = 0.1) in DMF at 90 °C for 24 h (G1, Table 2). Characteristic protons from Gem (see peaks *m*, *n* and *o* in Fig. S10†) were clearly visible in the ^1^H-NMR spectrum, resulting in a drug loading of 2.7 wt%. Similar rROPISA experiments were carried out in the presence of BMDO (*f*_BMDO,0_ = 0.66), with *f*_GemMA,0_ = 0.033 (G2) and 0.067 (G3). The expected structures were obtained, as assessed by ^1^H-NMR (Fig. S10†), with ∼10–12 mol% BMDO units inserted and drug loadings of 3.1 *vs.* 10 wt%, respectively. In terms of the degradation of the nanoparticle dry extracts, while G1 showed no decrease in *M*_*n*_ after degradation under accelerated conditions due to the absence of BMDO, G2 and G3 showed a clear shift in SEC traces towards lower *M*_*n*_ values, representing a −56 and −68% decrease in *M*_*n*_, respectively (Fig. S11†).

**Table 2:** Macromolecular characteristics of POEGMA-*b*-P(LMA-*co*-BMDO-*co*-GemMA) diblock copolymers

Ref.	[GemMA] : [LMA] : [BMDO]	Conv.^a^ (%)	*F*_BMDO_ b	*F*_GemMA_ b	DL_Gem_^c^ (wt%)	*M*_*n*_,_SEC_^d^ (g mol^−1^)	*M*_w,SEC_ d (g mol^−1^)	*Đ* d	*M*_*n*,exp_ after degradation^d^ (g mol^−1^)	*M*_w,exp_ after degradation^d^ (g mol^−1^)	*M*_*n*_ decrease^e^ (%)	*M*_w_ decrease^e^ (%)
G1	0.1 : 0.9 : 0	69	0	0.029	2.7	23 800	34 500	1.45	23 400	34 100	−2	−1
G2	0.1 : 0.9 : 2	76	0.10	0.033	3.1	21 600	39 600	1.88	9600	20 700	−56	−48
G3	0.2 : 0.8 : 2	69	0.12	0.116	10.0	14 600	29 500	2.02	4700	14 900	−68	−49

a LMA conversion determined by ^1^H-NMR, by integrating the two oxymethylene protons of LMA (5.5 and 6.0 ppm) and PLMA (3.8 ppm).

b Molar fraction of BMDO and Gem in the solvophobic block determined by ^1^H-NMR, by integrating the 4H of BMDO (7.1–7.5 ppm), excluding 1H from Gem and the 2H of LMA units (3.8–4.0 ppm).

c Drug loading in Gem determined by ^1^H-NMR, according to MW_Gem_/*M*_*n*,copolymer_, with MW_Gem_ = molecular weight of Gem and *M*_*n*,copolymer_ = *M*_*n*_ of the polymer prodrug considered.

d Determined by SEC after purification by dialysis.

e *M*_*n*_ decrease after the degradation of copolymers under accelerated conditions, calculated according to (exp. *M*_*n*,SEC_ − initial *M*_*n*,SEC_)/initial *M*_*n*,SEC_.

To demonstrate the relevance of the prodrug approach during this process, a control rROPISA similar to G1 was also carried out but in the presence of free Gem (5 wt%) instead of GemMA. As expected, no Gem was found in the copolymer nanoparticles after purification, as shown by the absence of characteristic Gem proton signals in the ^1^H-NMR spectrum (Fig. S12†). This result therefore ruled out potential adsorption of Gem onto the copolymer nanoparticles and the need to establish a chemical linkage between the drug and the copolymer.

**Fig. 5:**
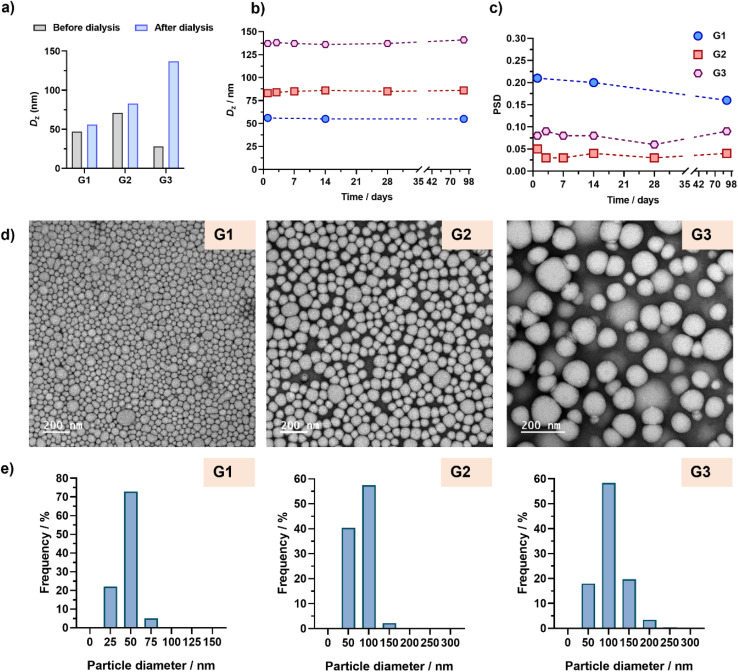
(a) Intensity-weighted mean diameters (*D*_*z*_) of POEGMA_28_-*b*-P(LMA-*co*-CKA-*co*-GemMA) copolymer nanoparticles G1–G3 determined by DLS in DMF (grey bars) and after dialysis against water (blue bars). Evolution with time of (b) *D*_*z*_ and (c) the particle size distribution (PSD) of nanoparticles G1–G3 after dialysis against water. (d) Representative TEM images and (e) particle size distributions (*n* = 450–1000) of nanoparticles G1–G3 determined by TEM (*d*_*n*_, *d*_w_, *d*_*z*_, and polydispersity values are given in Table S3†).

Remarkably stable aqueous suspensions of Gem-based polymer prodrug nanoparticles were successfully obtained in all cases over a period of more than 3 months. Interestingly, nanoparticles G1 and G2 showed similar sizes before and after dialysis (∼60–80 nm), in contrast to nanoparticles G3 (130 nm), presumably because of the high Gem content, as also observed with PtxMA (Fig. 5a and Table S2†). Importantly, insertion of BMDO units in the copolymer backbones did not affect the colloidal stability of nanoparticles in water (Fig. 5b and c). As shown by TEM, monodispersed spherical nanoparticles were obtained with average diameters matching the DLS data (Fig. 5d and e, Table S3†).

**Fig. 6:**
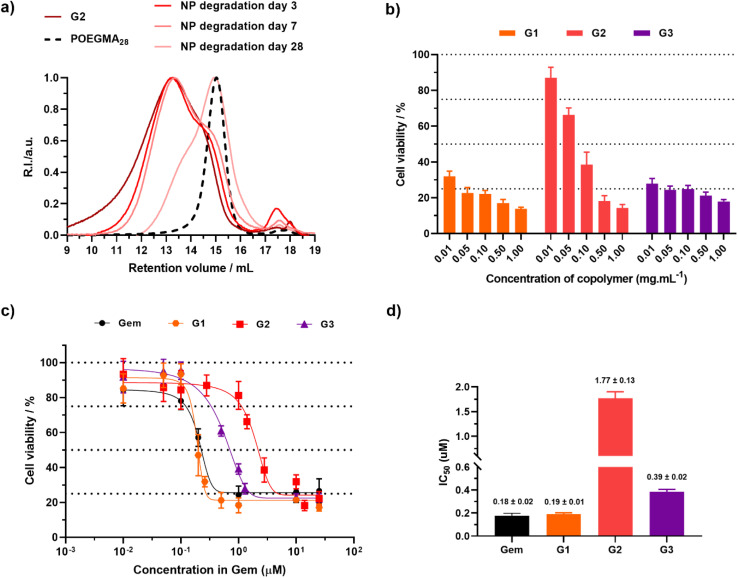
(a) SEC chromatograms after degradation of copolymers and nanoparticles G2 under accelerated conditions. The dashed line represents the SEC traces of the corresponding POEGMA_28_ macro-CTA. (b) Cell viability (MTT assay) expressed in copolymer mass concentration after incubation of A549 cells with increasing concentrations of nanoparticles G1–G3 for 72 h. Results are expressed as the percentage of absorption of treated cells ± SD in comparison with untreated cells (control). (c) Cell viability (MTT assay) expressed in Gem molar concentration and (d) corresponding IC_50_ values after incubation of A549 cells with nanoparticles G1–G3 for 72 h. Results were expressed as the percentage of absorption of treated cells ± SD in comparison with untreated cells (control). A One way ANOVA and an unpaired *t*-test were performed on PRISM 8.0.2 software. G1*vs.*G2*p* value = 0.0049 (**), *vs.*G3*p* value = 0.0583 (ns).

Degradation of nanoparticles G2 under accelerated conditions (Fig. 6a), led to a 26% decrease in *M*_*n*_ after 3 days, reaching 69% after nearly a month, similar to the Ptx-based counterparts (PT2).

Similar to Ptx-based copolymer prodrug nanoparticles, the cytotoxicity of POEGMA_28_-*b*-P(LMA-*co*-BMDO-*co*-GemMA) nanoparticles was then evaluated on A549 cancer cells and expressed in terms of copolymer mass concentration and gemcitabine molar concentration (Fig. 6b–d). G1–G3 nanoparticles clearly showed high toxicity in A549 cancer cells as early as 0.01–0.1 mg mL^−1^, in contrast to the drug-free nanoparticles C0, leading to 70% cell viability at 1 mg mL^−1^ (Fig. 6b). While free drugs usually lead to much lower IC_50_ values than the corresponding polymer prodrugs, due to the necessary time for their release while free drugs are immediately active, nanoparticles G1 and G3 exhibited IC_50_ values very close to those of free Gem (190–390 *vs.* 180 nM), demonstrating rapid and efficient release of the drug. Interestingly, the insertion of BMDO units in the copolymer seems to lead to higher IC_50_ values (190 *vs.* 390 nM), a trend that has already been observed with other types of polymer prodrug systems.^51^ The presence of BMDO is thought to increase the hydrophobicity of the polymer prodrug, leading to slower drug release.

### Cellular uptake of polymer prodrug nanoparticles

This new rROPISA process was then readily applied to the synthesis of fluorescent, degradable, polymer prodrug nanoparticles to confer therapeutic and imaging properties for potential theranostic applications. To fluorescently label nanoparticles, we chose to covalently link the fluorescent dye to the copolymer backbone instead of simply encapsulating it during the self-assembly process. This approach usually avoids dye leakage from nanoparticles and misinterpretation of fluorescence images with regard to nanoparticle localization.^52^

**Fig. 7:**
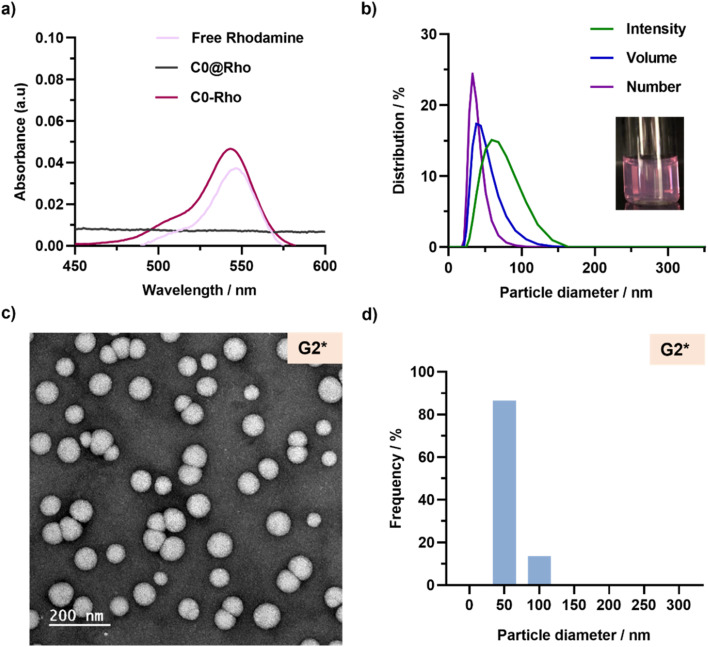
(a) Absorption spectra of free rhodamine B (Rho), P(OEGMA_24_-*co*-RhoMA)-*b*-P(LMA-*co*-BMDO) copolymer (C0-Rho) and POEGMA_28_-*b*-P(LMA-*co*-BMDO) copolymer (C0@Rho). (b) DLS measurements of nanoparticles G2*, giving average diameters expressed in intensity, volume and number. Inset: picture of the aqueous suspension of nanoparticle G2*. (c) Representative TEM image and (d) particle size distributions (*n* = 750) of nanoparticles G2*.

We selected a commercially available rhodamine B-functionalized methacrylate monomer (RhoMA) for RAFT-mediated copolymerization (*f*_RhoMA_ = 0.1 mol%) with OEGMA to fluorescently label the solvophilic block. After purification to remove unreacted RhoMA and OEGMA, the resulting P(OEGMA_24_-*co*-RhoMA) macro-CTA exhibited a strong purple color and well-defined characteristics (*M*_*n*_ = 8900, *Đ* = 1.08, Table S1†). It was then chain-extended under rROPISA conditions identical to those of C0, to produce fluorescent and degradable drug-free nanoparticles (C0-Rho). After purification, they exhibited a strong purple color with a fluorescent absorption signal around 545 nm (Fig. 7a) characteristic of rhodamine B, and an average diameter of 129 nm with a very low PSD (Table S4†). Importantly, the *in situ* physical encapsulation of free rhodamine B was also attempted under similar conditions (C0@Rho), but it did not result in a detectable amount of rhodamine B (Fig. 7a). These results confirmed the need for a covalent bond between rhodamine B and the copolymer backbone to produce fluorescent, degradable polymer prodrug nanoparticles.

Chain extension of the P(OEGMA_24_-*co*-RhoMA) macro-CTA was then successfully carried out with LMA, BMDO and GemMA (LMA : BMDO : GemMA = 0.9 : 2 : 0.1, G2*), or PtxMA (LMA : BMDO : PtxMA = 0.95 : 2 : 0.05, PT2*), to produce fluorescent, degradable polymer prodrug nanoparticles after dialysis. Similar colloidal characteristics were obtained compared with their non-fluorescent counterparts (Table S4†). For instance, nanoparticles G2* showed an average diameter of 59 nm with a PDI of 0.11 from DLS (Fig. 7b) and TEM images showed spherical nanoparticles with a similar size to that obtained by DLS (Fig. 7c and d, Table S3†).

**Fig. 8:**
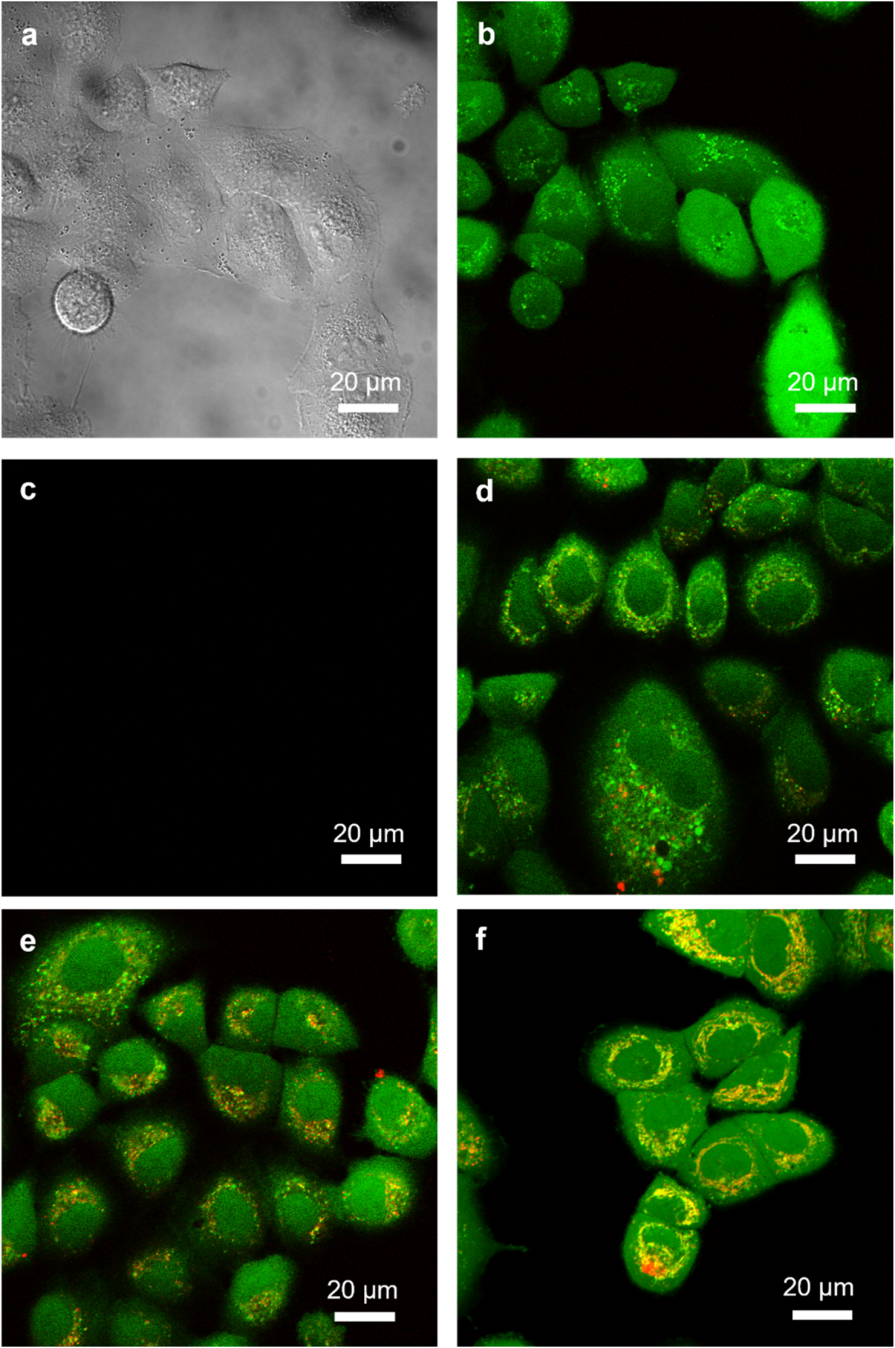
(a) Nomarski image of untreated A549 cells. (b and c) Confocal microscopy images of untreated A549 cells: (b) green (calcein AM) channel (*λ* = 505–550 nm) and (c) red (rhodamine) channel (*λ* = 585–700 nm). (d–f) Confocal microscopy images of A549 cells after incubation (d) with nanoparticles G2* for 2 h, (e) with nanoparticles G2* for 4 h and (f) with nanoparticles PT2* for 4 h. Pictures were taken from the median plane of cells. Scale bar = 20 μm.

Live-cell imaging was conducted by confocal microscopy to monitor the internalization of nanoparticles by A549 cancer cells, which is a relevant model for both Gem and Ptx (Fig. 8). A549 cancer cells were first treated with calcein-AM to stain the cell cytoplasm (green channel, *λ* = 505–550 nm), while the red channel (*λ* = 585–700 nm) was selected to track fluorescently-labeled nanoparticles PT2* and G2* after incubation with the cells for 2 or 4 h. While untreated cells exhibited only a green signal (Fig. 8a–c), cellular internalization of the fluorescent nanoparticles G2* occurred, as evidenced by the presence of yellow colocalization spots inside cells (Fig. 8d and e), resulting from the overlay between the green (calcein-AM staining) and red (nanoparticles) channels. The longer the incubation period, the greater the amount of internalized nanoparticles. Clear internalization of Ptx-based nanoparticles PT2* was also observed (Fig. 8f), showing the applicability of these nanoparticles to different anticancer drugs. Overall, these results demonstrated the potential of these degradable polymer prodrug nanoparticles to deliver anticancer drugs to cancer cells.

## Conclusion

We reported the successful development of high solid content and degradable polymer prodrug nanoparticles by the combination of rROP and PISA. This approach was easily applied to two different CKA monomers and two different anticancer drugs, opening the door to imaging/theranostic applications owing to the fluorescent labeling of the nanoparticles.

The nanoparticles were narrowly dispersed and remarkably stable in water. They were hydrolytically degraded under accelerated conditions, leading to significant cytotoxicity in cancer cells, even approaching the cytotoxicity of the free drug, demonstrating an efficient release of their payload. The discrete fluorescence labeling of their shell also enabled efficient monitoring of their fate by confocal microscopy and potential theranostic applications.

Owing to the structural diversity of vinyl polymers and the possibility to make them degradable, their ease of synthesis, particularly *via* reversible deactivation radical polymerization methods, and the robustness of the PISA process, we believe these new degradable nanoparticles could have the potential to challenge traditional polymer nanoparticles, especially those based on aliphatic polyesters or synthetic polypeptides.

An improvement to the system could focus on the use of a more environmentally friendly solvent than DMF, or on the use of thionolactones instead of CKAs to enable aqueous rROPISA^30–32^ and to facilitate the purification of polymer prodrug nanoparticles. Further developments could also be directed towards combination chemotherapy, by incorporating two types of drugs with different mechanisms of action. However, certain difficulties could arise, particularly in terms of characterization and control of drug composition, if there are excessive differences in steric hindrance and/or solubility between the two drug-bearing monomers.

## Data availability

Data will be made available on request.

## Author contributions

J. N. conceived and designed the research. J. N., C. Z. H. B., J. M. and M. L. designed the experiments. C. Z. H. B., J. M. and M. L. performed the experiments and analyzed the data. J. N. and C. Z. wrote the paper. All authors discussed the results and edited the paper.

## Conflicts of interest

The authors declare no conflict of interest.

## Supplementary Material

SC-016-D4SC07746F-s001
