# Supplementary material for: In situ synthesis of degradable polymer prodrug nanoparticles
Source: Chem Sci. 2025 Jan 7;16(6):2619–33. doi: 10.1039/d4sc07746f (PMC11733764; doi:10.1039/d4sc07746f)
Supplement: SC-016-D4SC07746F-s001 [file SC-016-D4SC07746F-s001.pdf]

Supplementary Information (SI) for Chemical Science.
This journal is © The Royal Society of Chemistry 2025

Supplementary Information

In Situ Synthesis of Degradable Polymer Prodrug Nanoparticles

*Chen Zhu, Hannah Beauseroy, Julie Mougin, Maëlle Lages, Julien Nicolas**

Université Paris-Saclay, CNRS, Institut Galien Paris-Saclay, 91400 Orsay, France

*To whom correspondence should be addressed.

Email: julien.nicolas@universite-paris-saclay.fr

Tel.: +33 1 80 00 60 81

† This article is dedicated to the memory of Dr. Maëlle Lages (08/21/2024)

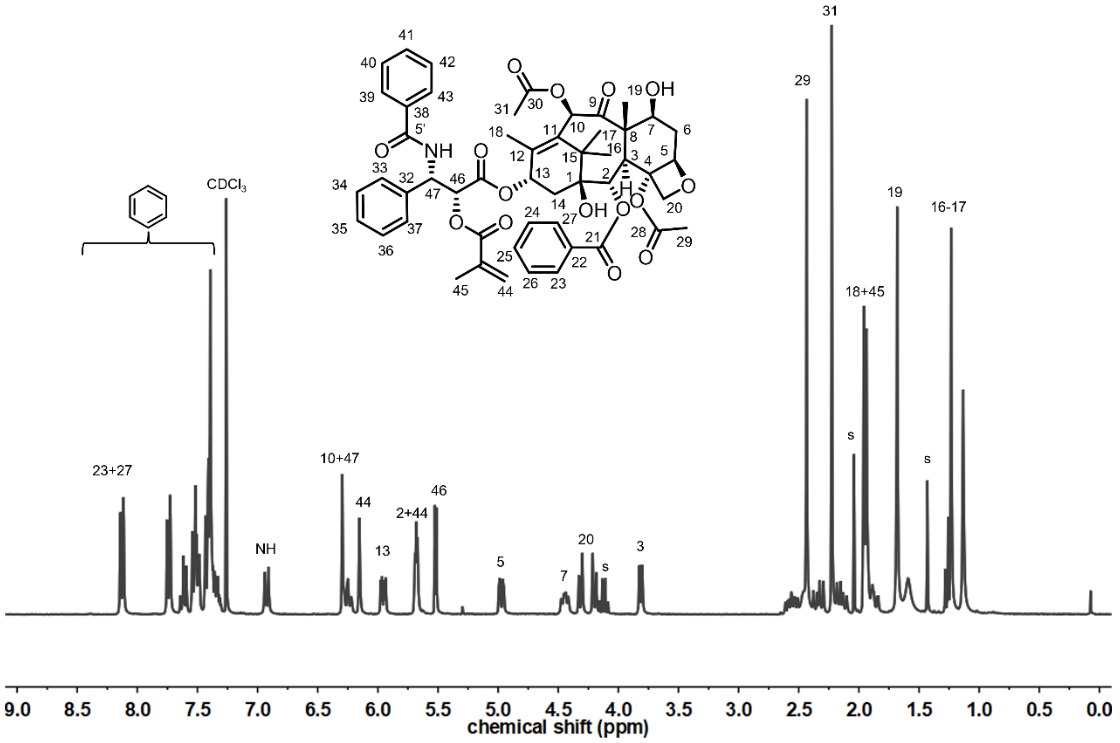

Figure S1. ^{1}H-NMR (300 MHz, $CDCl_3$) spectrum in the 0–9 ppm region of paclitaxel methacrylate (PtxMA). S (solvents) represent ethyl acetate and cyclohexane remaining traces.

Table S1. Macromolecular Characteristics of Poly[oligo(ethylene glycol) methyl ether methacrylate] (POEGMA) and P(OEGMA-*co*-RhoMA) Macro-Chain Transfer Agent Synthesized by RAFT Polymerization of OEGMA (and RhoMA) in Acetonitrile at 70 °C for 5 h.

**Macro-CTA**	**Targeted DP_n[a]**	**Conv.[b] (%)**	**$DP_{n,SEC}$[c]**	**$M_{n,SEC}$[d] (g.mol^{-1})**	**Đ[d]**	**$DP_{n,NMR}$[e]**	**$M_{n,NMR}$[e] (g.mol^{-1})**
**$POEGMA_{28}$**	50	49	28	8 900	1.10	28	8 800
**P($OEGMA_{24}$-*co*-RhoMA)**	50	43	28	8 900	1.08	24	7 600

[a] Calculated at 100% OEGMA conversion. [b] OEGMA conversion, determined by ^{1}H-NMR by integrating the two oxymethylene protons of OEGMA (4.3 ppm) and POEGMA (4.1 ppm). [c] Calculated by SEC according to $DP_{n,SEC} = (M_{n,SEC} - MW_{CDSPA}) / MW_{OEGMA}$. [d] Determined by SEC after precipitation. [e] Determined by ^{1}H-NMR by integrating the 18H of C_9H_{18} (1.2–1.4 ppm) and the 2H of POEGMA (4.1 ppm). DP_n is used to calculate $M_{n,NMR}$.

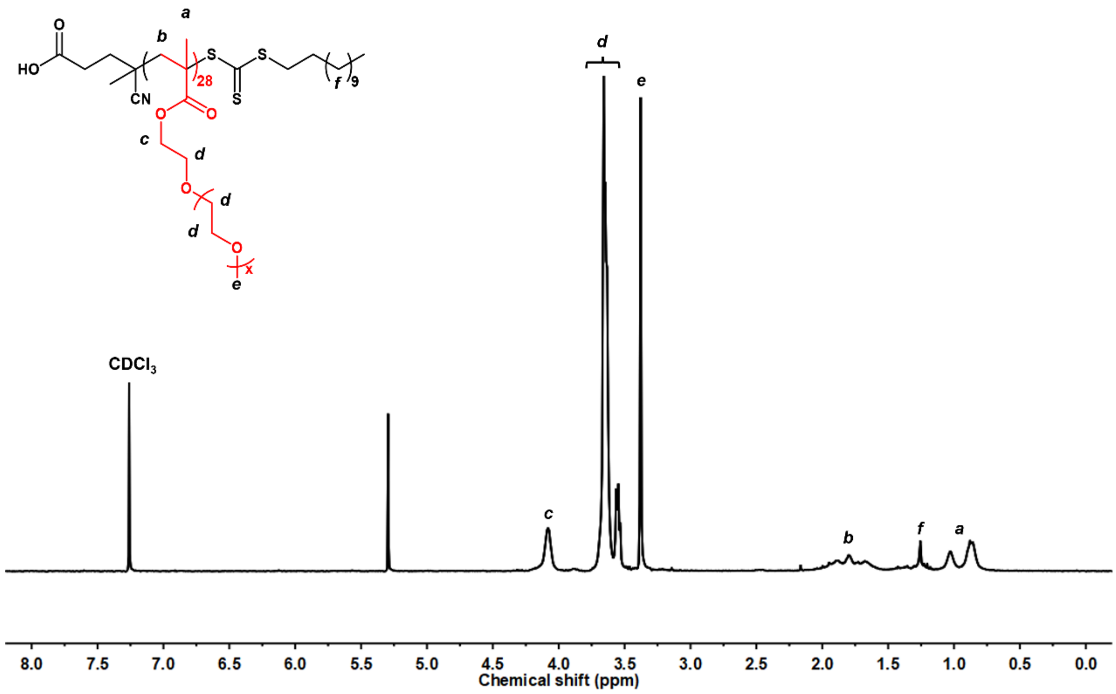

Figure S2. ^{1}H-NMR (300 MHz, $CDCl_3$) spectrum in the 0–8 ppm region of $POEGMA_{28}$ macro-CTA.

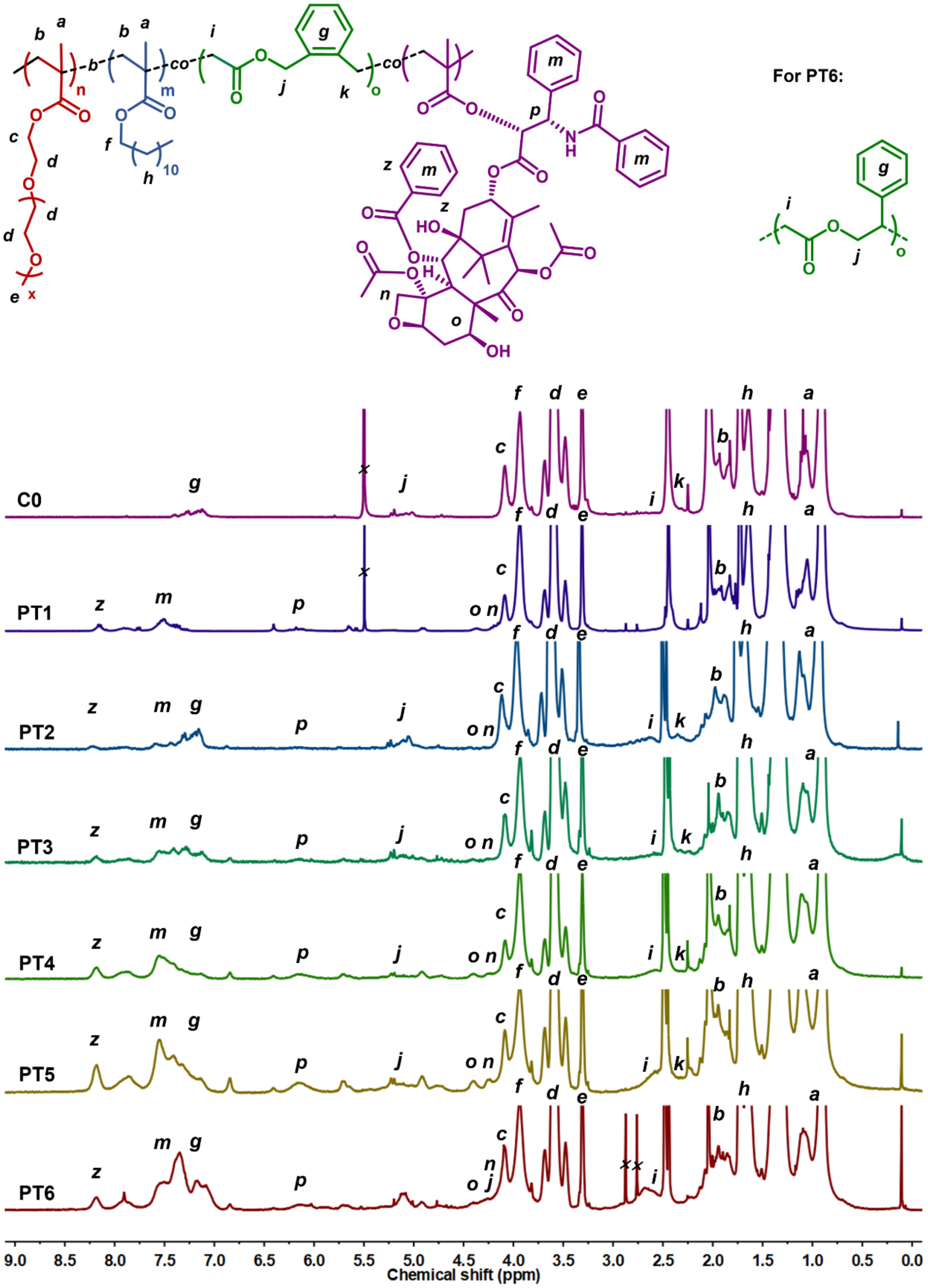

Figure S3. ^{1}H-NMR (300 MHz, TDF) spectra in the 0–9 ppm region of POEGMA$_{28}$-*b*-P(LMA-*co*-CKA-*co*-PtxMA) copolymers (CKA = BMDO for **PT1–5** and MPDL for **PT6**) after purification.

Table S2. Macromolecular and Colloidal Characteristics of POEGMA$_{28}$-*b*-P(LMA-*co*-CKA-*co*-PtxMA) and POEGMA$_{28}$-*b*-P(LMA-*co*-BMDO-*co*-GemMA) Copolymer Nanoparticles in DMF and water.

Ref.	D_z[a] (nm)	PSD[a]	D_z[b] (nm)	PSD[b]
**C0**	77	0.02	77	0.03
**PT1**	48	0.06	62	0.16
**PT2**	57	0.03	108	0.09
**PT3**	62	0.10	66	0.07
**PT4**	125	0.14	180	0.24
**PT5**	17	0.21	225	0.25
**PT6**	138	0.20	174	0.14
**G1**	47	0.16	56	0.20
**G2**	71	0.08	83	0.05
**G3**	28	0.31	137	0.10

[a] Determined by DLS in DMF. [b] Determined by DLS after dialysis.

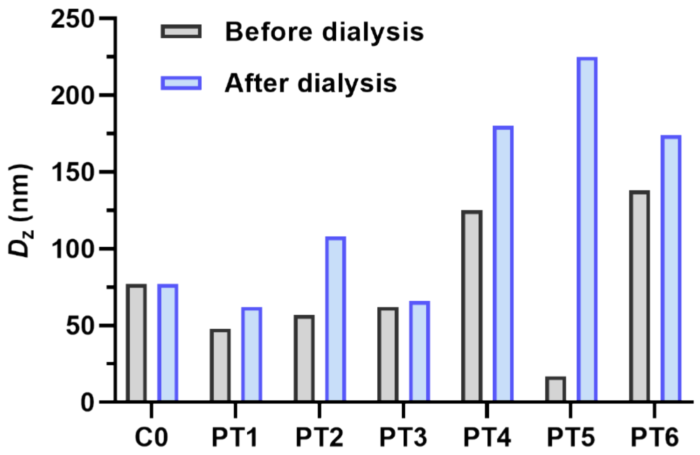

Figure S4. Intensity-average diameters (D_z) of: POEGMA$_{28}$-*b*-P(LMA-*co*-CKA-*co*-PtxMA) copolymer nanoparticle in DMF (grey bars) and after dialysis in water (blue bars). CKA = BMDO for **PT1–5** and MPDL for **PT6**.

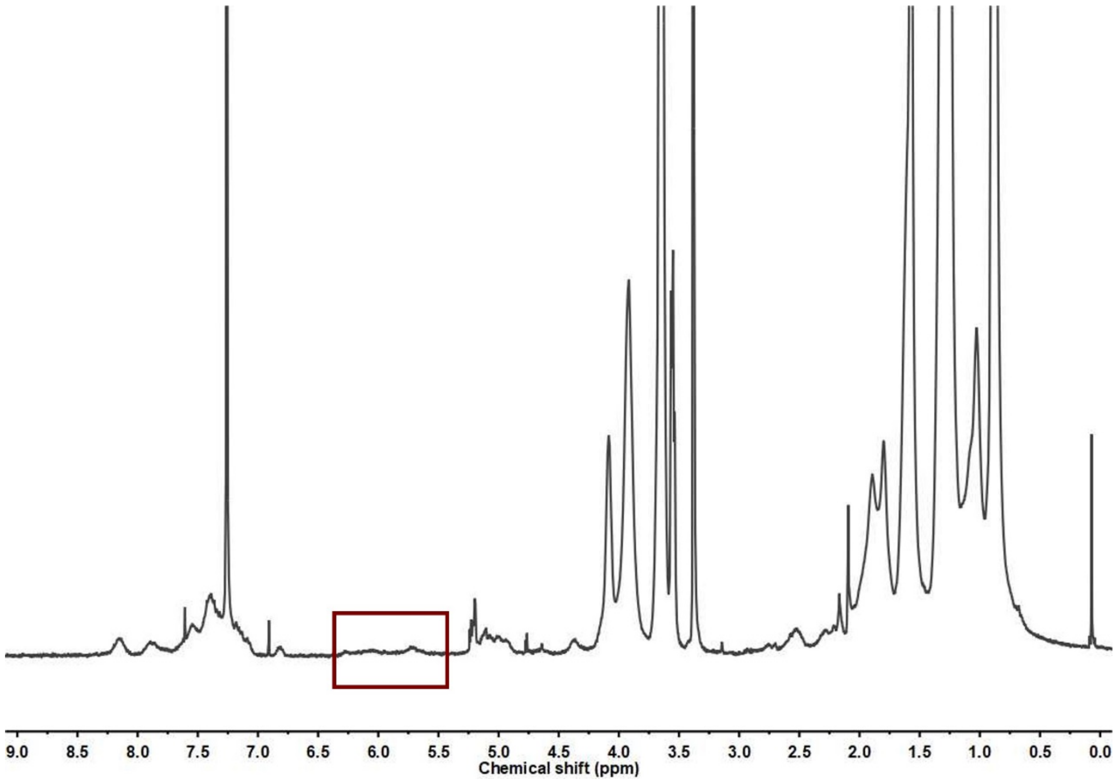

Figure S5. ^{1}H-NMR (300 MHz, $CDCl_3$) spectrum in the 0–9 ppm region of dried $POEGMA_{28}$-*b*-P(LMA-*co*-BMDO-*co*-PtxMA) copolymer nanoparticle **PT3** after dialysis in water. The red square indicates no observation of vinylic proton signals from remaining unreacted monomer.

Table S3. Transmission Electron Microscopy Data of $POEGMA_{28}$-*b*-P(LMA-*co*-CKA-*co*-PtxMA), $POEGMA_{28}$-*b*-P(LMA-*co*-BMDO-*co*-GemMA) or P($OEGMA_{24}$-*co*-RhoMA)-*b*-P(LMA-*co*-BMDO-*co*-GemMA) Copolymer Nanoparticles.

Ref.	DL[a] (wt %)	d_n[b] (nm)	d_w[b] (nm)	d_z[b] (nm)	PDI[b]
**C0**	0	71	82	98	1.15
**PT1**	11	54	60	67	1.11
**PT2**	3	94	111	130	1.18
**PT3**	13	77	85	93	1.10
**PT4**	19	70	81	90	1.16
**PT5**	33	86	107	143	1.25
**PT6**	20	93	100	107	1.08
**G1**	2.7	73	89	100	1.21
**G2**	3.1	84	92	101	1.10
**G3**	10.0	111	137	173	1.24
**G2***	3.6	72	81	94	1.13

[a] Drug loading determined by ^{1}H-NMR, according to: MW_{Drug} / $M_{n,NMR}$, with MW_{Drug} = molecular weight of the drug considered and $M_{n,NMR}$ = M_n of the polymer prodrug considered. [b] Determined as follows (n = 350–1000):

$$d_n = \frac{\sum_i n_i \cdot d_i}{\sum_i n_i}, \quad d_w = \frac{\sum_i n_i \cdot d_i^4}{\sum_i n_i \cdot d_i^3}, \quad d_z = \frac{\sum_i n_i \cdot d_i^6}{\sum_i n_i \cdot d_i^5}$$

and polydispersity index (PDI) = d_w / d_n.

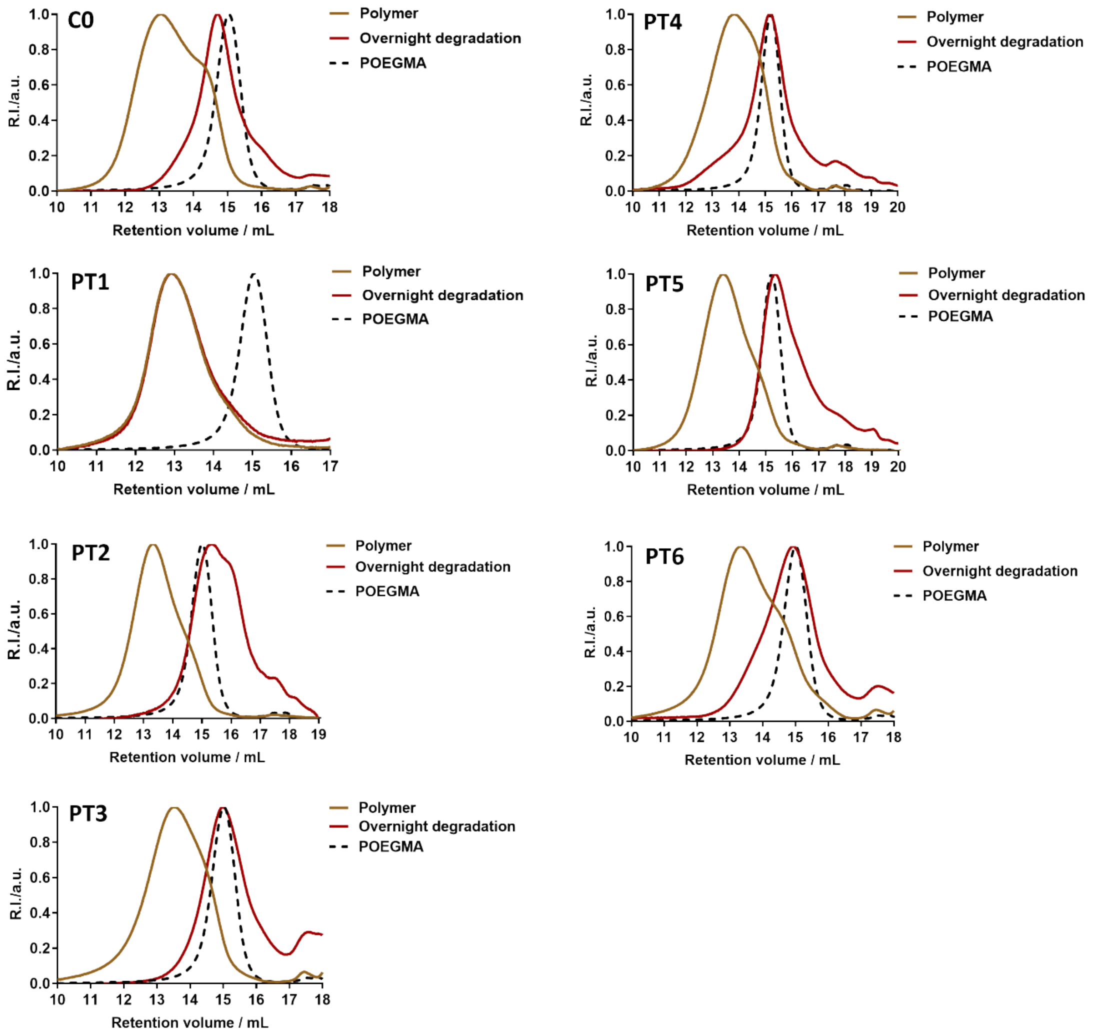

Figure S6. SEC chromatograms after overnight degradation of POEGMA$_{28}$-*b*-P(LMA-*co*-CKA-*co*-PtxMA) copolymers under accelerated conditions (THF/MeOH, KOH 2.5 %). The dashed lines represent the SEC traces of the corresponding POEGMA macro-CTA and the y-axis represent the normalized RI values. CKA = BMDO for **PT1–5** and MPDL for **PT6**.

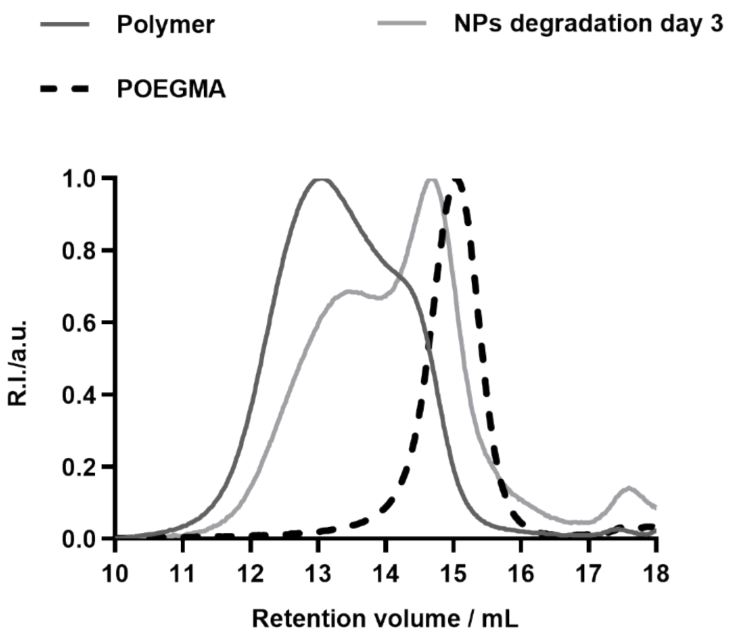

Figure S7. SEC chromatograms of $POEGMA_{28}$-*b*-P(LMA-*co*-BMDO) copolymer and nanoparticles **C0** after degradation under accelerated conditions (THF/MeOH, KOH 2.5 %).

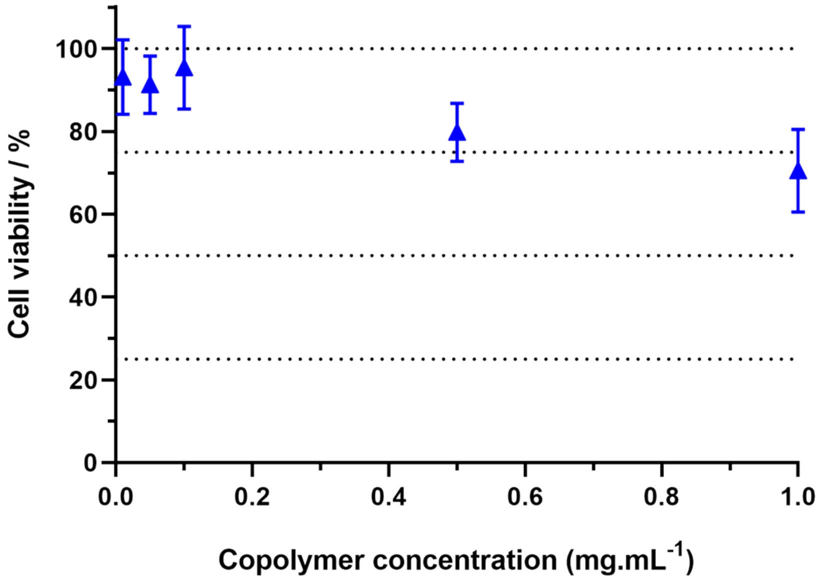

Figure S8. Cell viability (MTT assay) expressed in copolymer concentration after incubation of A549 cells with nanoparticles **C0** at different concentrations after 72h. Results were expressed as percentage of absorption of treated cells ± SD in comparison with untreated cells (control).

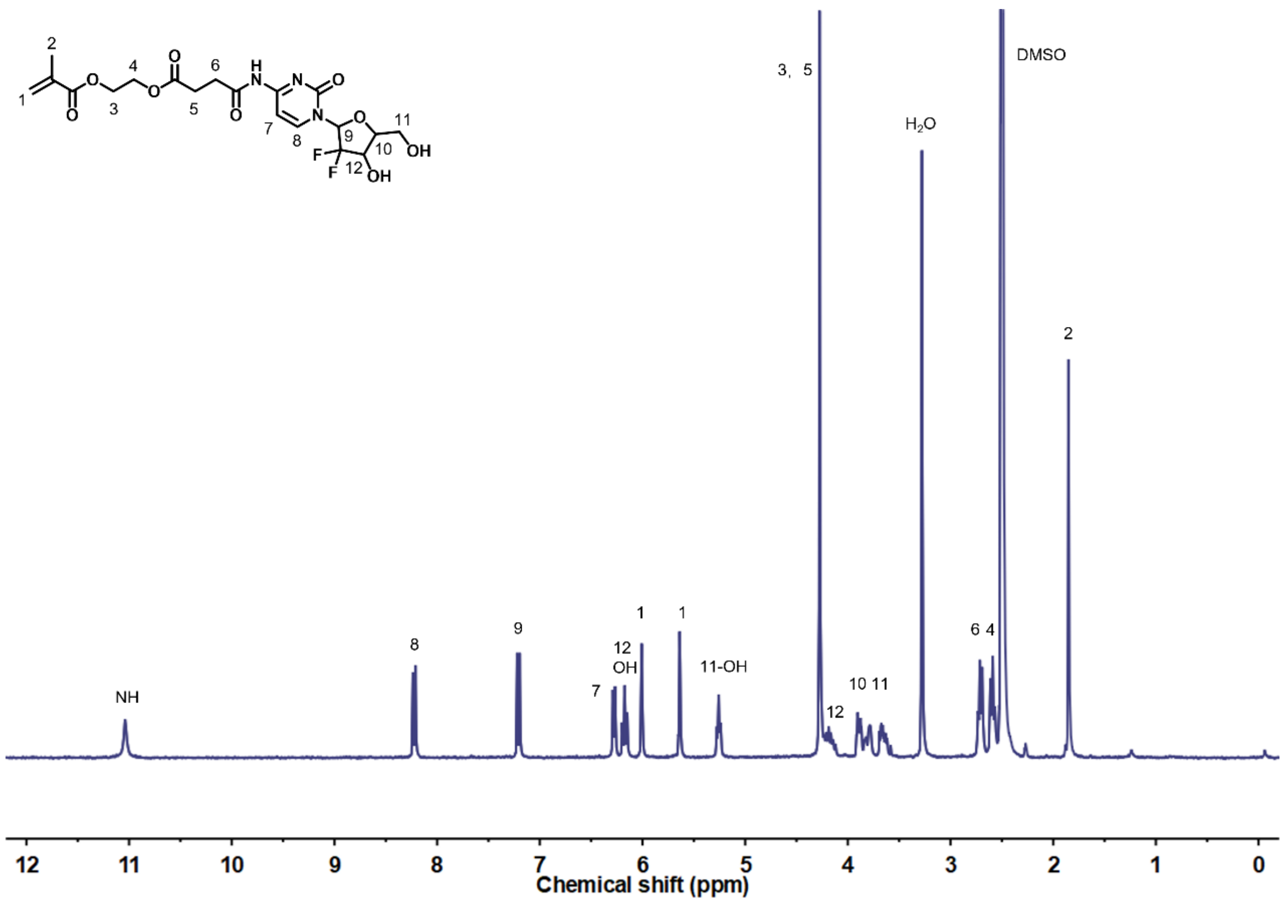

Figure S9. ^{1}H-NMR (300 MHz, DMSO-d_6) spectrum in the 0–12 ppm region of gemcitabine methacrylate (GemMA).

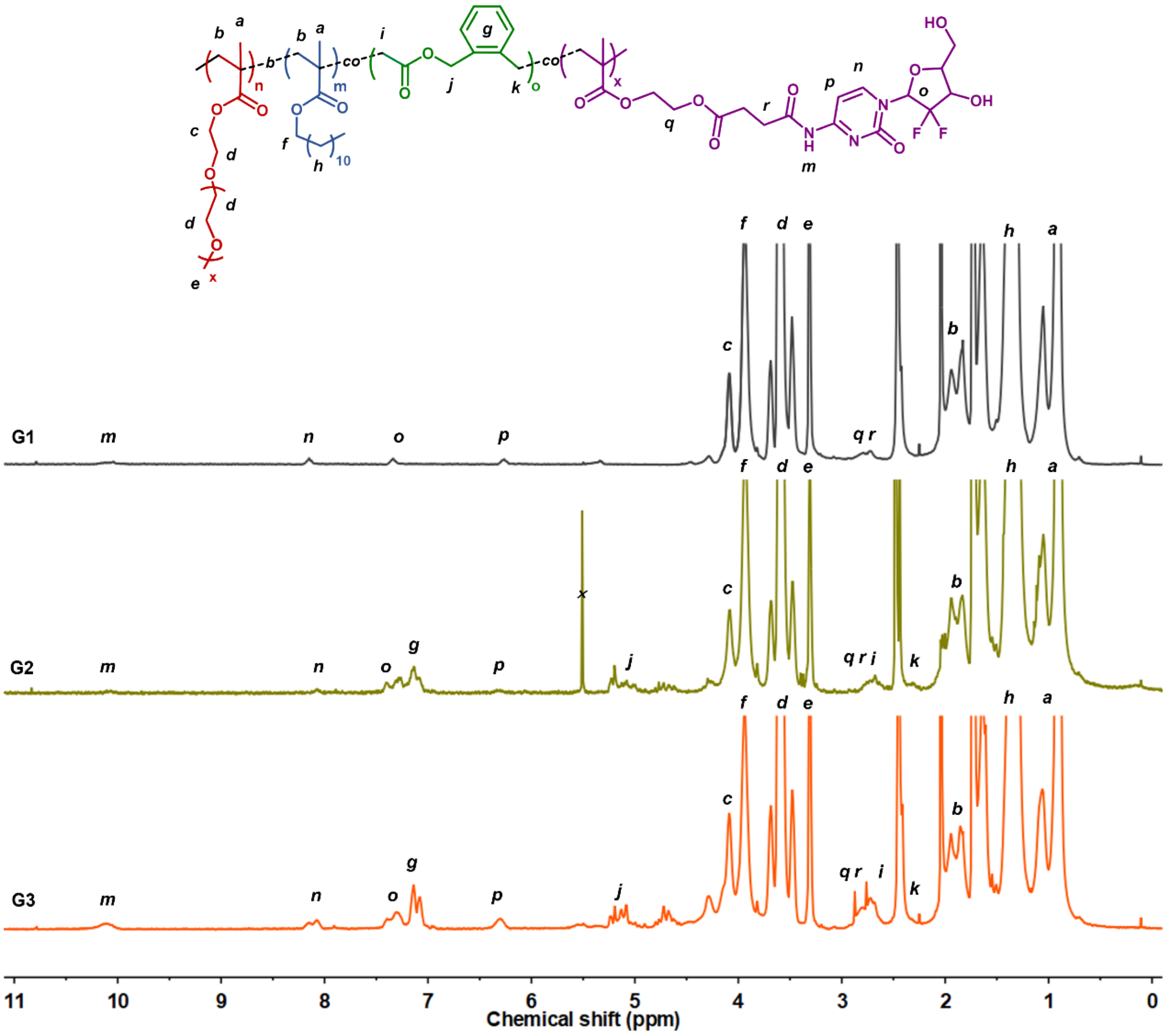

Figure S10. ^{1}H-NMR (300 MHz, TDF) spectra in the 0–11 ppm region of POEGMA$_{28}$-*b*-P(LMA-*co*-BMDO-*co*-GemMA) copolymers **G1–G3** after purification.

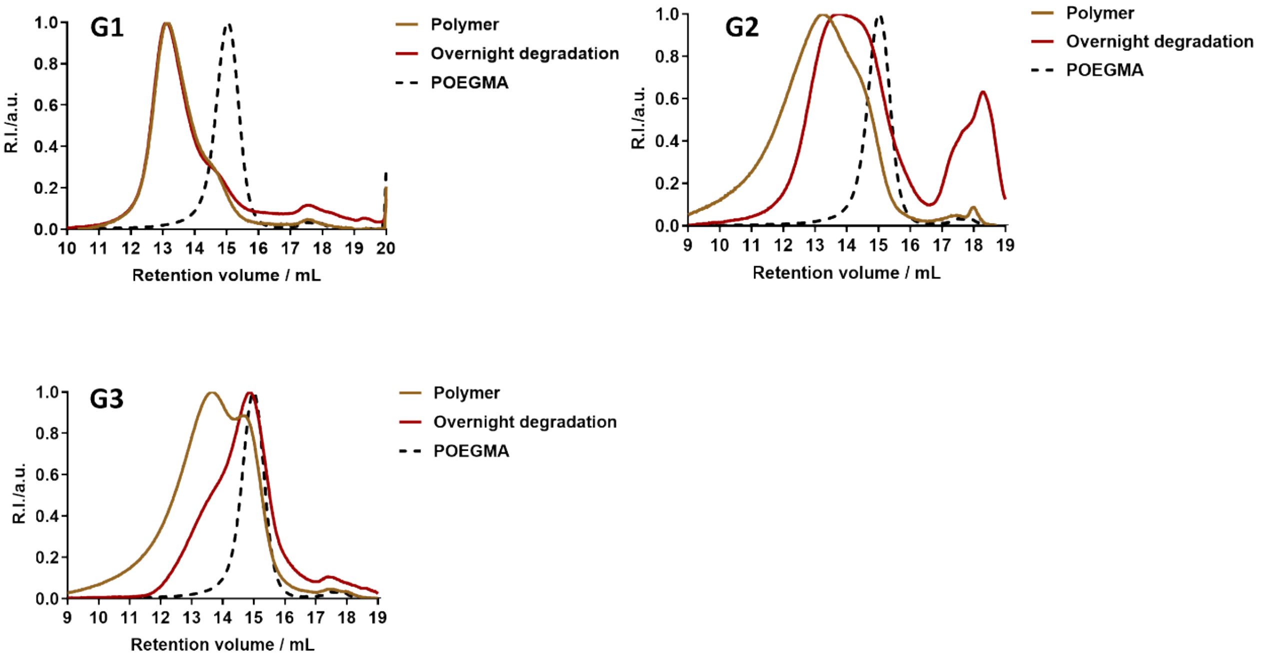

Figure S11. SEC chromatograms after overnight degradation of POEGMA$_{28}$-*b*-P(LMA-*co*-BMDO-*co*-GemMA) copolymers under accelerated conditions (THF/MeOH, KOH 2.5 %). The dashed lines represent the SEC traces of the corresponding POEGMA macro-CTA and the y-axis represent the normalized RI values.

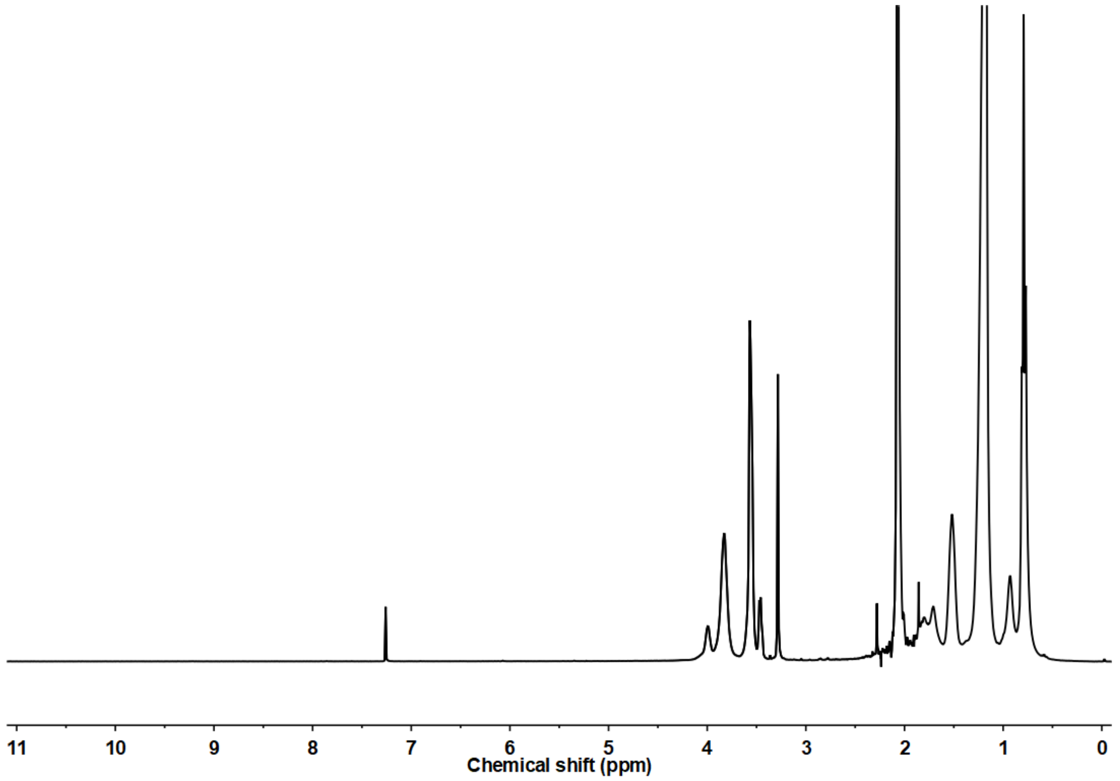

Figure S12. ^{1}H-NMR (300 MHz, $CDCl_3$) in the 0–11 ppm region of the dried copolymer after purification and after attempted in situ encapsulation of the free Gem.

Table S4. Macromolecular Characteristics of P(OEGMA-*co*-RhoMA)-*b*-P(LMA-*co*-BMDO) (**C0-Rho**), P(OEGMA-*co*-RhoMA)-*b*-P(LMA-*co*-BMDO-*co*-PtxMA) (**PT2***) and P(OEGMA-*co*-RhoMA)-*b*-P(LMA-*co*-BMDO-*co*-GemMA) (**G2***) diblock copolymer nanoparticles.

Ref.	Conv.[a] (%)	F_{CKA}[b]	DL[c] (wt%)	$M_{n,SEC}$[d] (g.mol^{-1})	$Đ$[d]	D_z[e] (nm) DMF	PSD[e]	D_z[e] (nm) water	PSD[e]	M_n decrease[f] (%)
**C0-Rho**	68	0.07	0	31 800	1.51	120	0.02	129	0.01	-74
**PT2***	74	0.08	4.0	12 500	2.08	145	0.12	131	0.08	-75
**G2***	70	0.11	3.6	19 300	1.69	58	0.12	59	0.11	-

[a] LMA conversion determined by ^{1}H-NMR by integrating the two oxymethylene protons of LMA (5.5 and 6.0 ppm) and PLMA (3.8 ppm). [b] F_{CKA} in the solvophobic block determined by ^{1}H-NMR by integrating the 4H of the BMDO aromatic ring (7.1–7.5 ppm) and the 2H of LMA units (3.8–4.0 ppm), after excluding the protons from drug. [c] Drug loading in Gem determined by ^{1}H-NMR, according to: MW_{Gem} / $M_{n,NMR}$, with MW_{Gem} = molecular weight of Gem and $M_{n,NMR}$ = M_n of the polymer prodrug considered. [d] Determined by SEC after dialysis. [e] Determined by DLS. [f] M_n decrease after degradation of copolymers under accelerated conditions, calculated according to: (exp. $M_{n,SEC}$ – initial $M_{n,SEC}$) / initial $M_{n,SEC}$.
